# Stagnating trends in complementary feeding practices in Bangladesh: An analysis of national surveys from 2004‐2014

**DOI:** 10.1111/mcn.12624

**Published:** 2018-07-12

**Authors:** Muzi Na, Víctor M. Aguayo, Mary Arimond, Anuradha Narayan, Christine P. Stewart

**Affiliations:** ^1^ Program in International and Community Nutrition, Department of Nutrition University of California Davis California USA; ^2^ Department of Nutritional Sciences, College of Health and Human Development The Pennsylvania State University Pennsylvania USA; ^3^ Nutrition Section, Programme Division United Nations Children's Fund (UNICEF) New York New York USA; ^4^ Center for Dietary Intake Assessment FHI 360 Washington, District of Columbia USA; ^5^ Nutrition Section United Nations Children's Fund (UNICEF) Bangladesh Country Office Dhaka Bangladesh

**Keywords:** Bangladesh, complementary feeding, demographic and health surveys, multilevel model, predictors, trends

## Abstract

Bangladesh has experienced steady socio‐economic development. However, improvements in child growth have not kept pace. It is important to document complementary feeding (CF) practices—a key determinant of children's growth—and their trends over time. The study aims to examine trends in CF practices in children aged 6–23 months using data from Bangladesh Demographic and Health Surveys conducted in 2004, 2007, 2011, and 2014. Multilevel logistic regression models were applied to identify independent predictors of four CF practice indicators among children 6–23 months, namely, timely introduction of complementary foods, minimum meal frequency, minimum dietary diversity, and minimum acceptable diet. Introduction of complementary foods was achieved among 64–71% of children between 2004 and 2014. The proportion meeting minimum meal frequency increased from 2004 to 2007 (71–81%) and declined and held steady at 65% from 2011 to 2014. The proportion meeting minimum dietary diversity in 2011 and 2014 was low (25% and 28%), and so was minimum acceptable diet (19% and 20%). From 2007 to 2014, child dietary diversity decreased and the most decline was in the consumption of legumes and nuts (29% to 8%), vitamin A‐rich fruits and vegetables (54% to 41%), and other fruits and vegetables (47% to 20%). Young child age (6–11 months), poor parental education, household poverty, and residence in the Chittagong and Sylhet independently predicted poorer feeding practices. Dietary diversity and overall diet in Bangladeshi children are strikingly poor. Stagnation or worsening of feeding practices in the past decade are concerning and call for decisive policy and programme action to address inappropriate child feeding practices.

**Key messages**

- Complementary feeding practices in Bangladesh were poor in 2004 and did not improve between 2004 and 2014.
- Child dietary diversity worsened from 2007 to 2014, with the largest declines observed in legumes and nuts (29% to 8%), vitamin A‐rich fruits and vegetables (54% to 41%), and other fruits and vegetables (47% to 20%).
- Complementary feeding practices varied by individual, household, and community characteristics. Young child age (6–11 months), poor parental education, household poverty, and residence in the Chittagong and Sylhet regions independently predicted poorer feeding practices, underscoring the need for policy and direct programme efforts to promote complementary feeding practices, especially for the youngest and most vulnerable children.

## 1 INTRODUCTION

Child malnutrition remains a serious problem in Bangladesh. A national survey in 2014 indicated that the proportion of children under five who were stunted, underweight, or wasted was 36%, 33%, and 14%, respectively (National Institute of Population Research and Training, Mitra and Associates, & ICF International, 2016). Historical data indicate that although the prevalence of child stunting and underweight has declined significantly, the annual rate of reduction has slowed down in more recent years. Under optimistic estimation, if the current rate of decline persists at ~3% point per year, more than 25% of children under five will still be stunted or underweight in 2025 (Government of the People's Republic of Bangladesh, 2017).

In recent years, Bangladesh has shown impressive economic growth. According to the World Bank Databank, between 2004 and 2014, the gross domestic product per capita in Bangladesh increased at an average annual rate of almost 5% (World Bank, 2017; see Figure S1). This strong economic growth has lifted many people out of poverty in both rural and urban areas (Figure S1a). Other socio‐economic and child health care indicators have also improved (Figure S1a), including primary education completion rate, coverage of improved water source and sanitation facilities, and women's empowerment.

However, socio‐economic development and health policy commitments do not seem to have solved the challenge of child undernutrition in Bangladesh. The current prevalence of stunting and underweight in 2014 remained unacceptably high at 36% and 33%, respectively (Figure S1c). The prevalence of child wasting (~14%), low birth weight (~22%), and anaemia in women (~44%) and children (~51%) have remained high, showing no significant change over the past decade (Figure S1c).

Though child malnutrition has multiple causes, it is widely agreed that inadequate infant and young child feeding (IYCF) is one of the most immediate determinants (Black et al., 2013; Stewart, Iannotti, Dewey, Michaelsen & Onyango, 2013). Rapid growth and critical development during infancy and early childhood require diverse, energy‐, and nutrient‐dense complementary foods that are introduced at the right time and fed with the right frequency and in right quantities (Arimond & Ruel, 2004; Black et al., 2013; Dewey, 2003; Frongillo et al., 2016; Horta & Victora, 2013). Child growth faltering is most evident from 3 to 24 months (Victora, de Onis, Hallal, Blössner, & Shrimpton, 2010), a window during which suboptimal feeding likely takes place, including disrupted exclusive breastfeeding in the first 6 months, early or delayed introduction of complementary foods, discontinued breastfeeding before age two, and inadequate quantity and/or quality of foods and feeding frequency. Because of the critical role of IYCF, several major nutrition programmes and policies in Bangladesh that aim to reduce child undernutrition have emphasized IYCF as a key component. These include the National Nutrition Programme (2006–2011), the National Nutrition Services (2011–2016), and the National Strategy on Prevention and Control of Micronutrient Deficiencies (2015–2024; Billah et al., 2017; Government of the People's Republic of Bangladesh, 2015; International Food Policy Research Institute, 2016).

However, complementary feeding (CF) practices among Bangladeshi children aged 6 to 23 months remain suboptimal and no national programmes exist to explicitly address this issue. In 2014, less than a quarter of children aged 6 to 23 months met the minimum criteria for acceptable diet (National Institute of Population Research and Training et al., 2016). Understanding the status of CF, how it is changing, and what factors predict poor CF practices will help identify unmet needs and populations at risk, thus informing the design and implementation of policies, programmes, and intervention strategies. The objective of this analysis is to document trends and predictors of CF practices in Bangladesh using repeated national survey data over a 10‐year period (2004–2014).

## 2 METHODS

### 2.1 Data source

We included in our analysis the latest four rounds of Bangladesh Demographic and Health Surveys (BDHS), conducted in 2004, 2007, 2011, and 2014, which included questions regarding complementary foods and feeding practices in children aged 6 to 23 months. Information about survey design, sample selection, training, and implementation can be found elsewhere (National Institute of Population Research and Training et al., 2016; National Institute of Population Research and Training, Mitra and Associates, & ICF International, 2013; National Institute of Population Research and Training, Mitra and Associates, & Macro International, 2009; National Institute of Population Research and Training, Mitra and Associates, & ORC Macro, 2005). Briefly, in each round the BDHS sample is a result of a two‐stage stratified sampling procedure. At the first stage, census enumeration areas (EAs), the primary sampling units (PSUs), were selected with probability proportional to the EA size. Both the 2004 and 2007 BDHS used the 2001 census and there were 361 PSUs selected at the first stage in both years. The 2011 and 2014 BDHS used the 2011 census of list of EAs and 600 PSUs were selected. At the second stage, within selected PSUs, households were selected using systematic sampling. On average, 30 households were selected per PSU. For the purpose of our analysis, information about CF practices and selected exploratory variables associated with CF practices were extracted from the women's interview and household interview datasets, which were available for public use. The household and women's response rate ranged between 97.9% and 99.8%. For the analytic sample, we included the youngest singleton children aged 6 to 23 months, who were alive at the time of survey and were living with their mothers.

### 2.2 Outcome measures

BDHS 2004 only included questions about timing of introduction of complementary foods and frequency of CF. BDHS 2007 included questions that assessed the consumption of several food groups during the previous 24 hr (day and night). In 2011 and 2014, BDHS included the World Health Organization (WHO) IYCF Module to assess IYCF practices in the previous 24 hr (day and night). We used the available data to calculate four population‐based CF indicators according to the WHO definitions (WHO, 2010).

In all four BDHS, respondents were asked to recall if the child ate any solid, semi‐solid, or soft foods in the previous 24 hr, during the day and at night. *Introduction of solid, semi‐solid, or soft foods (Intro)* was calculated as the proportion of infants 6–8 months of age who received complementary foods in the previous 24 hr.

Respondents were also asked to recall how often the child was fed solid, semi‐solid, or soft foods, and how many times the child consumed dairy products, including tinned, powdered or fresh animal milk, infant formula, and yogurt. For breastfed children, *Minimum meal frequency (MMF)* was calculated as a frequency ≥ 2 for children aged 6–8 months and ≥3 for children aged 9–23 months. For nonbreastfed children aged 6–23 months, MMF was calculated as a frequency ≥ 4, including the number of milk feeds. In 2004 and 2007, number of milk feeds was not available, therefore MMF could only be calculated for children who were breastfed.

In 2007, 2011, and 2014, respondents were asked to recall prelisted liquids and solids that the child consumed in the previous day and night while the interviewer coded their responses. The food items included in the questionnaire in each year are shown in Table S1. The food items were grouped into seven food groups, including grains, roots, and tubers; legumes and nuts; flesh foods; eggs; vitamin A‐rich fruits and vegetables; other fruits and vegetables; and dairy products. There were some differences in the IYCF module across years: in 2004, none of the individual food items were included; in 2007, flesh foods (e.g., meat and fish) and eggs were aggregated in one question and there was missing information about intake of baby cereal, tubers, vitamin A‐rich vegetables (e.g., pumpkin, carrots, and yellow squash), and other vegetables; in 2014, the “other vegetables” category was not included in the IYCF module. Given the disparity, we could only calculate minimum dietary diversity (MDD) in 2011 and 2014 knowing that incomplete data were used in the food group “other fruits and vegetables” in 2014. We also calculated a dietary diversity score based on six food groups after aggregating flesh foods and eggs. With acknowledgement of incomplete food item data in 2007 and 2014, we had data available for the six food groups in 2007, 2011, and 2014 for trend analysis of child dietary diversity.

Finally, if a breastfed child met MMF and MDD or a nonbreastfed child met MMF and was fed ≥2 milk feedings plus ≥4 food groups excluding milk products, we determined that the child had met the overall minimum requirement. We then calculated the proportion of children 6–23 months of age who met the overall minimum requirement in the previous day or night as *minimum acceptable diet (MAD)*. MAD was only calculated in 2011 and in 2014 with incomplete food group data.

### 2.3 Predictor variables

#### 2.3.1 Individual and household level

At individual level, we selected characteristics of the child, the mother, and/or the father that could be associated with child feeding and nutritional status (Black et al., 2013; Stewart et al., 2013). For children, child sex, breastfeeding status, age, birth order, birth interval, perceived birth weight, vitamin A supplementation in the previous 6 months, iron supplementation in children in the previous 7 days (not available in 2004 and 2007), vaccination status, and reported morbidity in the previous 2 weeks (diarrhoea, fever, cough) were considered. For mothers, we included age, body mass index (defined as weight in kilograms/squared height in meters), use of reproductive health care including place of delivery, type of delivery assistance, caesarean delivery, number of antenatal clinic visits, timing of postnatal check‐ups on women and child (not available in 2004), highest education level completed, marital status, exposure to media (newspaper, radio, TV), involvement in decision making (regarding large purchases for the households, freedom to visit family and friends, woman's own health care), and attitude towards domestic violence (whether beating wife is justified if she goes out without telling husband, neglects the children, argues with husband, refuses to have sex with husband, or burns the food). A composite women's empowerment score was calculated using an established coding scheme of available items under the “decision making” and “domestic violence” module (Jennings et al., 2014; Na, Jennings, Talegawkar, & Ahmed, 2015). For fathers, we included age, highest education level, and occupation. At the household level, we selected household head sex, number of household members, number of children under 5 years of age, types of cooking fuel, water (source of drinking water, location of water source, time to get water) and sanitation (toilet facility and whether toilet facility was shared) characteristics, and a precomposed wealth index using available socio‐economic variables and principal components analysis (Rutstein, & Johnson, 2004).

#### 2.3.2 Community level

The community level was defined at the PSU or cluster level. Place of residence, region, proportion of women completing primary or higher education, and mean women's empowerment score were used to describe the distribution of attributes among the eligible clusters, in which the mother–child dyads lived. In addition, we created a summary index of community‐level access to health care using all available data. The detailed algorithm to compose the index and methods to categorize five quintiles has been described previously (Na, Aguayo, Arimond, & Stewart, 2017). In sum, eight variables were available in 2004 (timing of postnatal check up on children and child iron supplementation were not assessed), whereas nine variables were available in 2007 (child iron supplementation was not assessed), 2011, and 2014 (maternal iron supplementation was not assessed) for deriving the community‐level access to health care variable.

### 2.4 Statistical analysis

STATA/SE 14.1 (STATA Corporation, College Station, TX, USA) was used to analyse data. The “svy” command was used to adjust for sampling weights in calculating mean, median, and proportions at the population level. The average annual rate of increase was calculated to measure the geometric progression ratio, at which proportion changes constantly over the period between first and latest observed year. Nonparametric tests were performed to test trends in ordinal variables over year. To test if rates of change in CF indicators differed in subgroups of the sample, an interaction term was created between the group and year and was added to the logistic regression model for each feeding indicator. The slopes representing the linearized rates were estimated, plotted, and compared against each other by contrasting the marginal effects. Delta‐methods were used to determine statistical significance (Cameron & Trivedi, 2005).

We constructed multivariable models to identify independent predictors following several steps to determine the number of levels in the model variance structure and final variable selection. First, we constructed the year‐specific null model for each CF indicator with no predictors under a one‐ (individuals only) and a two‐level (individuals and clusters) variance structure. Likelihood ratio tests were used to compare between the two nested models to determine whether the two‐level model significantly increased the proportion of explained variance for each CF indicator. With the exception of the Intro indicator in 2011, all tests resulted in a *p* value < .05 and we decided to apply the two‐level models in subsequent analysis. Second, we constructed year‐specific two‐level univariable models for each pair of predictor variable and CF indicator (Tables [Link], [Link]). Third, to construct year‐specific multivariable models for each CF indicator, we included all predictive variables with *p* < .1 in the univariable models as the initial step. We then removed variables with variance inflation factor (VIF) greater than 5, starting from the variable with the largest VIF, one by one until all VIF < 5. Finally, we pooled the data across years (2004, 2007, 2011, and 2014 for Intro and MMF; 2011 and 2014 for MDD and MAD) and included the combined set of predictors remaining in the final year‐specific multivariable analyses, and reran the analysis with the pooled (all years) data. After removing variables with VIF > 5, we finalized the two‐level multivariable models for Intro, MMF, MDD, and MAD, respectively, in the pooled analysis. Trends and odds ratios were considered statistically significant at *p* < .05. Sensitivity analyses were performed to check robustness of multilevel models by comparing results from (a) multilevel models using stepwise selection at *p* = .1 level; (b) multilevel models with child sex, maternal, and paternal age fixed as covariates; and (c) multilevel models with and without the year variable and models including year as a continuous or a categorical variable.

## 3 RESULTS

### 3.1 Trends in characteristics in study sample and clusters

Child, maternal, paternal, and household characteristics are presented in Table 1. The weighted numbers of eligible children in the study sample were 1793, 1721, 2334, and 2429, respectively, in 2004, 2007, 2011, and 2014. The majority of children aged 6–23 months were breastfed; this declined slightly from 2004 to 2014 (96% to 94%, *p* trend < .001). There were also declines in the proportion of children with reported diarrhoea (12% to 8%, *p* trend < .001), fever (49% to 42%, *p* trend < .01), or cough (52% to 37%, *p* trend < .001), and a declining prevalence of maternal undernutrition (BMI < 18.5 kg/m^2^; 43% to 27%, *p* trend < .001). The prevalence of maternal overweight or obesity, however, increased three folds from 5% to 15% (*p* trend < .001). The proportion of mothers completing at least primary education increased by 20% points from 66% to 86% (*p* trend < .001). The proportion of mothers watching TV increased slightly from 44% to 49% (*p* trend < .05). Indicators of women's empowerment improved by 10–24% points (all *p* trends < .05). The proportion of fathers completing primary or higher education increased slightly (27% to 30%, *p* trend < .001). At the household level, the proportion with unimproved sources of drinking water remained low (3.6% to 2.8%, *p* trend = .39), whereas there was a significant reduction in the prevalence of households with unimproved toilet facilities (44% to 31%) and shared toilets (45% in 2007 to 35%, both *p* trend < .001).

**Table 1:** Trends of individual‐, household‐, and community‐level demographic and socio‐economic characteristics in study sample and clusters from 2004 to 2014 in Bangladesh

		2004		2007		2011		2014		AARI	*P* trend
		*N*	% or mean (SE)	*N*	% or mean (SE)	*N*	% or mean (SE)	*N*	% or mean (SE)		
*N*		1,793		1,721		2,334		2,429			
*Child characteristics*											
Female		1,793	51.3	1,721	50.1	2,334	49.3	2,429	47.5	−0.8	.06
Currently breastfed		1,793	96.4	1,721	95.8	2,334	94.4	2,429	93.5	−0.3	***
Age (months)		1,793		1,721		2,334		2,429			.68
6–11			32.2		35.5		36.1		34.9	0.8	
12–17			40.1		30.5		34.8		34.1	−1.6	
18–23			27.6		34.0		29.1		30.9	1.1	
Birth order		1,793		1,721		2,334		2,429			***
Firstborn			30.7		36.8		36.0		39.9	2.7	
Second to fourth			54.7		53.1		55.5		54.1	−0.1	
Fifth and more			14.6		10.1		8.5		6.1	−8.5	
Child health: had the following symptom in the past 2 weeks											
Diarrhoea		1,791	12.3	1,721	13.9	2,333	7.5	2,428	7.9	−4.3	***
Fever		1,793	48.7	1,721	45.6	2,333	45.0	2,427	42.0	−1.5	**
Cough		1,793	51.8	1,720	44.4	2,333	41.8	2,428	37.2	−3.2	***
*Maternal characteristics*											
Age (years)		1,793	24.2 (0.17)	1,721	24.0 (0.16)	2,334	24.2 (0.13)	2,429	24.3 (0.14)		.44
BMI (kg/m^2^)		1,774		1,709		2,287		2,429			***
Undernourishment (<18.5)			43.2		35.9		33.1		26.5	−4.8	
Normal (18.5–24.9)			52.2		57.4		58.2		58.7	1.2	
Overweight or obesity (≥25)			4.6		6.7		8.7		14.8	12.5	
Primary or higher education completed		1,793	65.5	1,721	77.9	2,334	83.3	2,429	86.4	2.8	***
Exposure to media: at least once a week											
Reading newspaper		1,793	7.5	1,721	6.4	2,333	5.3	2,421	5.9	−2.4	.06
Listening to radio		1,793	33.7	1,721	19.1	2,333	4.8	2,429	2.4	−23.2	***
Watching TV		1,793	44.2	1,721	45.5	2,333	47.6	2,429	49.4	1.1	*
Involved in decision making on											
Large household purchases		1,653	47.8	1,561	54.1	2,309	54.5	2,395	57.3	1.8	***
Visiting family and friends		1,660	46.8	1,553	56.6	2,306	57.2	2,395	58.8	2.3	***
Regarding own health care		1,715	39.1	1,590	54.0	2,309	59.5	2,395	63.3	4.9	***
*Paternal characteristics*											
Age (years)		1,764	33.6 (0.22)	1,685	32.9 (0.24)	2,301	32.8 (0.17)	2,392	32.5 (0.17)		***
Primary or higher education completed		1,792	26.5	1,718	27.8	2,332	29.9	2,428	30.0	1.3	***
*Household characteristics*											
Female household head		1,793	6.2	1,721	9.6	2,334	7.6	2,429	9.0	3.8	.12
No. of household members		1,793	6.5 (0.09)	1,721	6.4 (0.11)	2,334	6.2 (0.08)	2,392	6.1 (0.08)	‐	***
No. of children under 5 years		1,793	1.5 (0.02)	1,721	1.4 (0.03)	2,334	1.4 (0.03)	2,429	1.3 (0.02)	‐	***
Unimproved source of drinking water		1,793	3.6	1,721	2.8	2,334	1.5	2,429	2.8	−2.4	.39
Unimproved toilet facility		1,793	43.9	1,721	62.3	2,334	47.0	2,429	31.4	−3.3	***
Shared toilet with other households		1,793	N/A	1,721	45.4	2,334	39.7	2,429	35.3	−3.5	***

	2004		2007		2011		2014			
*Community characteristics*	*N* or % or median	[IQR]	*N* or % or median	[IQR]	*N* or % or median	[IQR]	*N* or % or median	[IQR]	AARI	*P* trend
No. of clusters	361		356		584		582		‐	
Rural residence	66.2		62.9		66.1		65.8		−0.1	.77
Geographical region										**
Barisal	11.9		13.8		12.0		11.7		−0.2	
Chittagong	17.7		17.4		15.8		15.6		−1.2	
Dhaka	23.5		21.9		17.8		18.4		−2.4	
Khulna	15.0		14.9		14.4		13.6		−1.0	
Rajshahi	22.2		18.8		28.3		28.9		2.7	
Sylhet	9.7		13.2		11.8		11.9		2.0	
Access to health care										
% Children completed age‐appropriate vaccine	71.4	[58.8, 81.3]	80.0	[66.7, 90.0]	83.3	[71.4, 90.0]	81.8	[69.2, 90.9]	1.4	***
% Delivered at health facility	7.7	[0.0, 18.8]	11.8	[5.0, 31.0]	27.0	[10.0, 44.9]	40.0	[18.2, 66.7]	17.9	***
% Delivered with professional assistance	10.5	[5.0, 25.0]	15.8	[6.5, 36.4]	30.0	[11.8, 50.0]	42.9	[22.2, 70.0]	15.1	***
% Had caesarean delivery	0.0	[0.0, 7.1]	6.3	[0.0, 16.2]	11.8	[0.0, 25.0]	20.0	[0.0, 40.0]		***
% Had ≥4 ANC visits	13.3	[4.3, 30.0]	17.9	[6.7, 38.5]	21.4	[8.3, 44.4]	28.6	[13.3, 50.0]	8.0	***
% Postnatal check‐up on woman within 1 day of delivery	7.1	[0.0, 15.8]	26.8	[14.3, 43.3]	25.0	[9.1, 44.4]	41.7	[20.0, 57.1]	19.4	***
% Postnatal check‐up on child within 1 day of delivery	N/A	N/A	25.0	[11.8, 44.1]	38.0	[20.0, 60.0]	37.5	[12.5, 54.5]		**
% Children 0–5 years received vitamin A in the last 6 months	70.6	[60.0, 80.0]	77.8	[66.7, 86.2]	57.1	[40.0, 72.7]	61.5	[45.5, 73.3]	−1.4	***
% Children 0–5 years received iron pills, sprinkles, or syrup in the last 7 days	N/A	N/A	N/A	N/A	0.0	[0.0, 0.0]	0.0	0 [0.0, 7.7]	‐	***
% Women given or bought iron tablets during pregnancy	53.8	[35.7, 69.2]	58.3	[41.2, 75.0]	N/A	N/A	N/A	N/A	2.7	*

*Note*. *P* trend values are from linear or logistic regression between continuous or binary sample characteristics and year, adjusting for complex sampling design. *P* trend values for ordinal variables are from nronparametric tests. AARI = average annual rate of increase.

* *p* < .5;

** *p* < .01;

*** *p* < .001.

The distributions of community characteristics are presented for the 361, 356, 584, and 582 eligible clusters (Table 1). Around two‐thirds of the clusters were in rural areas (63–66%). The rate of child completed age‐appropriate vaccination increased from 71% to 82% and the utilization of reproductive health services and nutritional supplementation, including health facility delivery, professional assistance at delivery, Caesarean delivery, antenatal care, postnatal care, child vitamin A supplementation, and child and maternal iron supplementation, also increased significantly between 2004 and 2014 (all *p* trend < .001).

### 3.2 Trends in CF indicators

**Figure 1:**
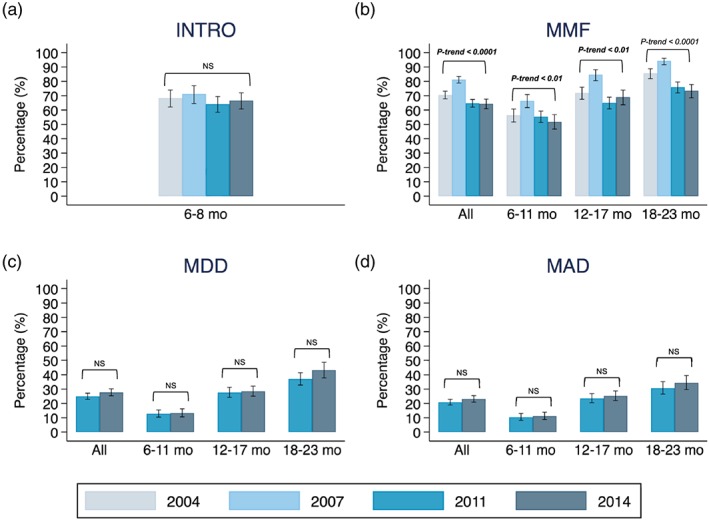
Complementary feeding in Bangladeshi children aged 6–23 months 2004–2014. (a) Introduction of solid, semi‐solid, or soft foods (INTRO); (b) minimum meal frequency (MMF); (c) minimum dietary diversity (MDD); and (d) minimum acceptable diet (MAD). Only the breastfed children were included in panel (b) and (d) due to missing data in number of milk feeds in 2004 and 2007

The proportion of children meeting the different CF indicators is presented by child age and year (Figure 1). Intro was achieved by 68%, 71%, 64%, and 67% of children aged 6–8 months in 2004, 2007, 2011, and 2014, respectively (*p* trend = .31). In the same years, the proportion of children aged 6–23 months who met MMF was 71%, 81%, 65%, and 65%, showing a decreasing trend regardless of child age group (all *p* trend < .001). The proportion of children meeting MDD was 25% and 28% in 2011 and 2014, respectively (*p* trend = .10), whereas the proportion of children meeting MAD was 19% and 20% in 2011 and 2014, respectively (*p* trend = .15). Among the three age groups, the proportion meeting MMF, MDD, and MAD was the lowest in children aged 6–11 months, among whom only 52%, 13%, and 11% met each minimum required criterion, respectively.

This general secular trend in CF indicators held true in other population subgroups with some differential changes over time (Figures S2–S4). For example, the proportion of children meeting the MMF criterion decreased over time but more so among children whose mothers had no education (Figure S2, difference in slope *p* < .05) and among children living in the poorest households (Figure S3, difference in slope *p* < .01) as compared with the better‐off reference groups. MDD and MAD were achieved in higher proportions in both 2011 and 2014 among children whose mothers had better education, who lived in wealthier households, and in communities with better access to health and nutrition care. The gaps associated with differences in community‐level access to health care narrowed over time (Figure S4).

### 3.3 Trends in food group consumption

**Figure 2:**
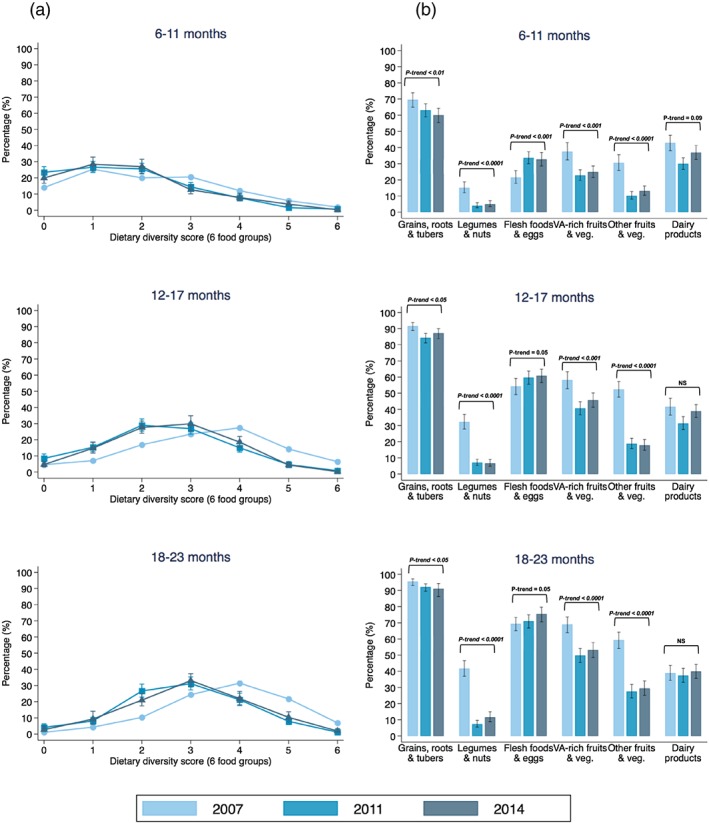
Child dietary diversity score based on six food groups (a) and food group consumption (b) in 2007–2014. VA = vitamin A

The distribution of child dietary diversity scores and the proportion of children consuming individual food groups are presented by child age (Figure 2). Among children aged 6–11 months, the weighted mean (95%CI) dietary diversity score was 2.2 (2.0, 2.3), 1.6 (1.5, 1.7), and 1.7 (1.6, 1.8) in 2007, 2011, and 2014. In the same years, the corresponding mean (95%CI) dietary diversity among children aged 12–17 months was 3.3 (3.2, 3.4), 2.4 (2.3, 2.5), and 2.6 (2.5, 2.7), whereas the corresponding values among children aged 18–23 months were 3.7 (3.6, 3.9), 2.9 (2.7, 3.0), and 3.0 (2.9, 3.1), respectively. In all three age groups, there was a significant declining trend in child dietary diversity over time (all three *p* trend < .001). The deterioration in food group diversity was most observable in legumes and nuts (percentage in 2007, 2010, and 2014, respectively: 29%, 6%, 8%), vitamin A‐rich fruits and vegetables (54%, 37%, 41%), and other fruits and vegetables (47%, 18%, 20%). The proportion consuming flesh foods was the same in 2011 and 2014 (44%). The proportion consuming eggs was also similar across the two survey years (23% and 26%, respectively; Figure S6).

### 3.4 Independent predictors of appropriate CF practices

The predictors included in the analysis of pooled data (all years) are presented for the four CF indicators in Table 2 (the year‐specific results are available in Tables [Link], [Link]). Compared with the youngest age group, the adjusted odds of meeting MMF, MDD, and MAD in children aged 12–17 months were 2–3 times higher and odds were 3–5 times higher for children aged 18–23 months. Maternal education independently predicted the likelihood of meeting MMF, MDD, and MAD, but not Intro, in a similarly dose‐responsive manner, with 21–26% lower odds among children of mothers with primary education and 29–46% lower odds among children of mothers without education, as compared with children of mothers with secondary or higher education. Compared with the referent children living in Barisal, children living in Sylhet had 24%, 28%, and 32% lower odds of meeting MMF, MDD, and MAD criteria, respectively; children in Chittagong had 35% (*p* = .06) and 31% lower odds of meeting Intro and MMF; whereas children in Rajshahi had 1.4–2 times higher odds of meeting Intro, MMF, and MAD, respectively.

**Table 2:** Independent predictors of meeting complementary feeding practice [OR(95%CI)] using multivariable multilevel logistic regression analysis

	Intro			MMF			MDD			MAD		
	Estimate		*P* value	Estimate		*P* value	Estimate		*P* value	Estimate		*P* value
	OR	(95%CI)		OR	(95%CI)		OR	(95%CI)		OR	(95%CI)	
*N*		1,402			7,693			4,543			4,498	
Year												
2004	1.00	(Referent)		1.00	(Referent)							
2007	1.06	(0.72, 1.58)	.76	1.96	(1.60, 2.39)	***						
2011	0.68	(0.47, 1.00)	*	0.75	(0.63, 0.89)	**	1.00	(Referent)		1.00	(Referent)	
2014	0.75	(0.51, 1.11)	.15	0.74	(0.62, 0.89)	**	1.12	(0.96, 1.31)	0.16	1.19	(1.01, 1.39)	*
*Child characteristics*												
Age (months)												
6–11				1.00	(Referent)		1.00	(Referent)		1.00	(Referent)	
12–17				2.11	(1.86, 2.39)	***	2.89	(2.38, 3.50)	***	2.82	(2.30, 3.45)	***
18–23				3.42	(2.96, 3.95)	***	4.63	(3.81, 5.64)	***	3.90	(3.17, 4.79)	***
Birth order												
Firstborn	0.85	(0.63, 1.15)	.29	1.13	(0.97, 1.31)	.12	1.33	(1.14, 1.56)	***	1.36	(1.15, 1.61)	***
Second to fourth	1.00	(Referent)		1.00	(Referent)		1.00	(Referent)		1.00	(Referent)	
Fifth and more	0.91	(0.57, 1.44)	.69	0.92	(0.74, 1.14)	.45	1.05	(0.75, 1.47)	.77	1.17	(0.82, 1.66)	.38
Birth interval (month)												
No previous birth												
<24				1.25	(1.02, 1.54)	*	1.28	(0.95, 1.72)	.10	1.50	(1.10, 2.03)	**
≥24				1.00	(Referent)		1.00	(Referent)		1.00	(Referent)	
Age‐appropriate vaccination												
None	0.98	(0.52, 1.85)	.95	0.93	(0.67, 1.30)	.69	0.91	(0.51, 1.62)	.76	0.76	(0.40, 1.45)	.41
Some	0.78	(0.58, 1.06)	.11	0.90	(0.78, 1.03)	.14	0.89	(0.72, 1.11)	.31	0.87	(0.69, 1.10)	.26
Complete	1.00	(Referent)		1.00	(Referent)		1.00	(Referent)		1.00	(Referent)	
*Maternal characteristics*												
Age (years)												
15–24				1.02	(0.87, 1.20)	.82						
25–34				1.00	(Referent)							
35–49				1.16	(0.96, 1.40)	.13						
BMI (kg/m2)												
<18.5	1.23	(0.93, 1.63)	.14	1.12	(0.99, 1.26)	.07	0.94	(0.79, 1.11)	.46	0.99	(0.82, 1.18)	.88
18.5–24.9	1.00	(Referent)		1.00	(Referent)		1.00	(Referent)		1.00	(Referent)	
≥25	0.66	(0.41, 1.08)	.10	0.95	(0.77, 1.17)	.63	1.15	(0.92, 1.44)	.23	1.15	(0.91, 1.45)	.23
Reproductive health care												
Type of delivery assistance												
Health professional	1.27	(0.90, 1.79)	.17	0.94	(0.81, 1.10)	.45	0.83	(0.69, 1.00)	.05	0.83	(0.69, 1.01)	.07
Traditional birth attendant	1.25	(0.79, 2.00)	.34	1.00	(0.82, 1.23)	.98	0.83	(0.64, 1.08)	.18	0.88	(0.67, 1.16)	.38
Other	1.00	(Referent)		1.00	(Referent)		1.00	(Referent)		1.00	(Referent)	
Antenatal clinic visits												
None	0.71	(0.52, 0.98)	*	0.81	(0.70, 0.93)	**	0.97	(0.78, 1.19)	.75	0.98	(0.79, 1.23)	.88
1–3	1.00	(Referent)		1.00	(Referent)		1.00	(Referent)		1.00	(Referent)	
≥4	1.05	(0.75, 1.47)	.79	1.05	(0.90, 1.21)	.54	1.22	(1.02, 1.45)	*	1.16	(0.97, 1.39)	.10
Education												
No education	0.68	(0.46, 1.03)	.07	0.71	(0.59, 0.86)	***	0.54	(0.40, 0.73)	***	0.57	(0.41, 0.78)	**
Primary	0.89	(0.64, 1.24)	.49	0.74	(0.64, 0.85)	***	0.71	(0.58, 0.86)	**	0.79	(0.65, 0.97)	*
Secondary or higher	1.00	(Referent)		1.00	(Referent)		1.00	(Referent)		1.00	(Referent)	
*Paternal characteristics*												
Age (years)												
<31	1.00	(Referent)		1.00	(Referent)							
≥31	0.98	(0.74, 1.32)	.92	1.17	(1.02, 1.33)	*						
Education												
No education	0.86	(0.59, 1.24)	.41	0.92	(0.78, 1.09)	.32	0.76	(0.59, 0.96)	*	0.74	(0.57, 0.95)	*
Primary	1.29	(0.92, 1.80)	.14	1.03	(0.89, 1.19)	.70	0.87	(0.73, 1.05)	.16	0.87	(0.71, 1.05)	.15
Secondary or higher	1.00	(Referent)		1.00	(Referent)		1.00	(Referent)		1.00	(Referent)	
*Household characteristics*												
Household wealth												
Richest	1.00	(Referent)		1.00	(Referent)		1.00	(Referent)		1.00	(Referent)	
Richer	0.87	(0.57, 1.34)	.54	0.95	(0.79, 1.15)	.60	0.92	(0.74, 1.15)	.47	0.95	(0.75, 1.20)	.66
Middle	1.16	(0.74, 1.82)	.52	0.95	(0.78, 1.17)	.65	0.67	(0.52, 0.86)	**	0.72	(0.56, 0.94)	*
Poorer	0.84	(0.53, 1.36)	.48	0.83	(0.67, 1.03)	.09	0.57	(0.43, 0.76)	***	0.58	(0.43, 0.78)	***
Poorest	0.95	(0.57, 1.57)	.84	0.91	(0.73, 1.14)	.43	0.52	(0.38, 0.71)	***	0.55	(0.40, 0.76)	***
*Community characteristics*												
Geographical region												
Barisal	1.00	(Referent)		1.00	(Referent)		1.00	(Referent)		1.00	(Referent)	
Chittagong	0.65	(0.42, 1.02)	.06	0.69	(0.56, 0.86)	**	0.81	(0.61, 1.08)	.16	0.81	(0.60, 1.10)	.18
Dhaka	1.28	(0.80, 2.05)	.31	1.10	(0.88, 1.38)	.41	0.95	(0.71, 1.28)	.75	1.14	(0.84, 1.54)	.39
Khulna	1.55	(0.87, 2.73)	.13	1.94	(1.49, 2.53)	***	1.21	(0.89, 1.66)	.23	1.49	(1.08, 2.06)	*
Rajshahi	2.04	(1.25, 3.33)	**	1.43	(1.15, 1.79)	**	1.24	(0.95, 1.63)	.12	1.38	(1.04, 1.83)	*
Sylhet	0.90	(0.55, 1.48)	.69	0.76	(0.60, 0.96)	*	0.72	(0.52, 0.99)	*	0.68	(0.48, 0.95)	*
Rank of access to health care												
Highest (best access)	1.00	(Referent)		1.00	(Referent)		1.00	(Referent)		1.00	(Referent)	
Higher	0.80	(0.50, 1.29)	.36	0.97	(0.78, 1.20)	.76	0.88	(0.69, 1.13)	.33	0.91	(0.71, 1.18)	.48
Medium	0.71	(0.44, 1.15)	.17	0.96	(0.78, 1.19)	.73	0.88	(0.68, 1.13)	.32	0.88	(0.68, 1.14)	.35
Lower	0.72	(0.44, 1.17)	.18	0.97	(0.78, 1.20)	.76	0.91	(0.70, 1.20)	.52	0.96	(0.73, 1.26)	.76
Lowest (worse access)	0.56	(0.33, 0.94)	*	0.89	(0.70, 1.12)	.32	1.14	(0.85, 1.54)	.37	1.14	(0.84, 1.55)	.40

*Note*. Intro = introduction of solid, semi‐solid, and soft foods; MMF = minimum meal frequency; MDD = minimum dietary diversity; MAD = minimum acceptable diet; OR = odds ratio; CI = confidence interval.

* *p* < .05;

** *p* < .01;

*** *p* < .001.

There were other predictors that were consistently associated with MDD and MAD but not Intro or MMF, including 33–36% lower odds of meeting MDD and MAD among children who were firstborn (comparing with second to fourth birth), 24–26% lower odds among children whose fathers had no education (comparing with secondary or higher education), and 5–8%, 28–33%, 42–43%, 45–48% lower odds among children living in the progressively poorer household wealth quintiles (comparing with the richest quintile).

## 4 DISCUSSION

Using nationally representative data from 2004 to 2014, we were able to document trends in CF practices in Bangladesh over the past decade. The proportion of children who were introduced to CF in a timely manner (Intro) remained at about two‐thirds in the last decade, whereas the proportion of children aged 6–23 months meeting MDD and MAD stagnated at around one quarter. A limitation in this analysis was the lack of data on other vegetables in 2014. However, there was no substantial change in the intake trend for all other plant‐source food groups between 2011 and 2014 (Figure 2), and therefore one would expect to see a similar proportion of other vegetables reported in 2014. Thus, the stagnation in MDD and MAD would likely remain even if other vegetable data in the 2014 BDHS were available. The proportion of children meeting MMF criterion and the mean child dietary diversity scores, however, worsened. Despite general steady improvements in economic, health, and living conditions, as indicated by national‐level socio‐economic indicators and our sample data, CF practices in children aged 6–23 months did not keep pace with these improvements.

The declining trend in MMF in Bangladesh was in contrast to the slow progress observed in Nepal over the same period of time from 68% in 2001 to 82% in 2014 (Na et al., 2018). More importantly, our analysis indicates that some vulnerable subpopulations, including children of mothers with no formal education and children from the poorest wealth quintile households, experienced a greater deterioration in MMF. It has been hypothesized that insufficient feeding frequency may be associated with lack of mothers' time to prepare food and to feed children. A study in the 1990s analysed panel data on time use of rural Bangladeshi women and food consumption of children under 2 and did not find evidence linking women's time and child calorie intake (Jain & Zeller, 2015). However, this relationship may have changed in the past 20 years, during which many women shifted from working on farms to off‐farm employment, such as in manufacturing garment factories (Kabeer & Mahmud, 2004) and in microfinance groups (Bangladesh Bank, 2002). A recent systematic review has revealed mixed findings linking microfinance participation to child nutritional outcomes (Orton et al., 2016). Programmes and initiatives to improve CF practices in Bangladesh need to pay attention to promoting and supporting increased meal feeding frequency. Particularly—but not exclusively—among disadvantaged subgroups of children whose mothers are less educated, children who live in poverty, and children who live in communities with poor access to health care and nutrition services.

Similarly, the apparent disparities in MDD and MAD call for policy and programme attention in all groups, particularly the most disadvantaged. Hierarchical separations in the probability of meeting MDD and MAD were clearly observed by levels of maternal formal education, household wealth, and community‐level access to health care. The community‐level access to health care was a proxy to evaluate potential spillover benefits beyond individual‐level utilization of health care comparing communities with high degree of access with communities with low degree of access. Between 2011 and 2014, such inequities remained unchanged between children of less educated and more educated mothers as well as between children from poorer and richer households. The consistent inequity differentials indicate that poor maternal education and lower socio‐economic status were the major constraints limiting children's food consumption, food diversity, and potentially nutritional outcomes. Recent data from the Alive and Thrive project in Bangladesh revealed that changes in underlying maternal education, socio‐economic status, and food security altogether accounted for 34% of the total change in child linear growth, which was independent from maternal knowledge in nutrition and feeding practices (Nguyen et al., 2017).

Our findings from the food consumption analysis and the analysis of predictors of poor CF have several implications for future interventions in improving CF practices in Bangladesh:

First, in line with previous studies in South Asia (Chowdhury, Rahman, & Khan, 2016; Na, Aguayo, Arimond, & Stewart, 2017), younger children aged 6 to 11 months should be given priority attention by policies, programmes, and initiatives as they are at a higher risk of not being fed appropriately in terms of MMF, MDD, and MAD.

Second, to promote child dietary diversity in poor households, several locally affordable nutrient‐rich foods could be promoted, including (a) legumes and nuts, which were fed to less than 10% of children; (b) vitamin A‐rich fruits and vegetables, which showed a declining trend in intake from 2007 to 2014 that could be partially driven by variation in survey months in 2007 (that covers the peak season of mangos in May and June) and 2011 and 2014 (from June–July to December. See Figure S5). In addition, due to missing information in vitamin A‐rich vegetables in 2007 and other vegetables in 2007 and 2014, fruit and vegetable consumption could have been underestimated in the corresponding years; (c) other fruits and vegetables, which also declined from ~50% in 2007 to ~22% in 2014 that may not entirely be explained by seasonality (Figure S5); and (d) eggs, which were fed to less than a third of children and even fewer among children aged 6–11 months. Recent evidence from Latin America shows the potential of introducing eggs early in children's diets both in increasing the intake of protein and improving linear growth in young children (Iannotti et al., 2017).

Third, future nutrition behaviour and social change education interventions should be continuously tailored to audiences with limited or no formal education. Successful nutrition education trials that have improved feeding practices and child growth, share characteristics of good design (e.g., visual tools including charts and posters), actionable messaging (e.g., home‐prepared recipes), standardized training and quality control, and frequent visits (Bhutta et al., 2013; Lassi, Das, Zahid, Imdad, & Bhutta, 2013). Tailored intervention design and planning facilitate that caregivers without formal education understand, are inspired, implement the intended behaviour‐change messages, and adopt appropriate/improved CF practices.

Fourth, feasible and simple indicators are needed for targeting programmes and resources towards communities where CF practices are poor. Though the community‐level access to health care indicator independently differentiated the odds of meeting only one CF indicator in Bangladesh, a similarly derived community‐level indicator strongly predicted the odds of meeting MDD and MAD criteria in Pakistan and Nepal (Na, Aguayo, Arimond, Dahal, et al., 2018; Na, Aguayo, Arimond, & Stewart, 2017). From an implementation perspective, community‐level interventions by trained community volunteers and health care workers have potential to reach vulnerable populations at scale. Intervention programmes such as those implemented by Alive and Thrive (Menon et al., 2016) and CARE (Owais et al., 2017) in Bangladesh that were delivered by trained community health and nutrition workers have been effective at improving CF practices in the intervention areas.

Finally, strategies to improve access to food need to be coupled with communication interventions for behaviour and social change, as financial, physical, and social constraints to optimal CF may coexist in Bangladeshi families (Manikam et al., 2017).

Our analysis has strengths and weaknesses. It was limited by the cross‐sectional nature of the data, with different sampling frames between recent and more remote survey years including some missing values. Therefore, our analysis can only identify associations and cannot determine causal relationships. The data available in the BDHS also limits our ability to examine all potential risk factors for poor CF at the individual, household, and community levels. The lack of information regarding access to infant and young child feeding‐specific services also prevented us from examining more direct health access indicators at individual and community level. However, our analysis has important strengths, including the use of nationally representative data over multiple years that were collected using a common and globally agreed upon methodology. The trend analysis over the past 10 years used nationally representative data and likely represented true distributions in the general population. Multivariable multilevel models were applied in the predictor analysis, the results of which were robust and were supported by a series of sensitivity analysis (data not shown). The standard methodology used for data analysis and presentation provides for potential comparability with previous and ongoing analyses across different countries in South Asia.

In sum, our analysis indicates stagnation or worsening trends in some of the key CF indicators in Bangladesh over the past decade. These worrying findings call for advocacy, policy, and programmatic efforts to reprioritize the improvement of complementary foods and feeding for infants and young children in the context of the larger national development agenda in Bangladesh.

## CONFLICTS OF INTEREST

The authors declare that they have no conflicts of interest.

## CONTRIBUTOR STATEMENT

MN, VMA, MA, and CPS conceptualized the research question. MN requested data, conducted literature review, data analysis, and prepared the first draft of the manuscript. VMA, MA, AN, and CPS provided technical support on study methods, insights on results interpretation, and revisions to the manuscript. All authors read and approved the final manuscript.

## Supporting information

Figure S1: Changes in (A) socio‐economic status; (B) child health indicators; and (C) maternal and child nutritional status between 2004 and 2014 in Bangladesh. Data is extracted from World Bank Databank, unless otherwise indicated: a. Lee 2013 and b. BDHS 2011
Figure S2: Proportion of meeting complementary feeding criteria by maternal education
Figure S3: Proportion of meeting complementary feeding criteria by household wealth
Figure S4: Proportion of meeting complementary feeding criteria by community‐level access to health care
Figure S5: Proportion of food group consumption in 2007, 2011 and 2014 by survey months
Figure S6: Proportion of flesh foods and eggs consumption in 2011 and 2014

Table S1: Questions about solid, semi‐solid, and soft foods given to the child in the last 24 hours in 2004, 2007, 2011, and 2014

Table S2: Factors [OR(95%CI)] in relation to Intro using year‐specific univariate multilevel logistic regression analysis

Table S3: Factors [OR(95%CI)] in relation to MMF using year‐specific univariate multilevel logistic regression analysis

Table S4: Factors [OR(95%CI)] in relation to MDD using year‐specific univariate multilevel logistic regression analysis

Table S5: Factors [OR(95%CI)] in relation to MAD using year‐specific univariate multilevel logistic regression analysis
