# Supplementary material for: Stagnating trends in complementary feeding practices in Bangladesh: An analysis of national surveys from 2004‐2014
Source: Matern Child Nutr. 2018 Jul 12;14(Suppl 4):e12624. doi: 10.1111/mcn.12624 (PMC6586058; doi:10.1111/mcn.12624)
Supplement: Supplementary file 1 — Figure S1: Changes in (A) socio‐economic status; (B) child health indicators; and (C) maternal and child nutritional status between 2004 and 2014 in Bangladesh. Data is extracted from World Bank Databank, unless otherwise indicated: a. Lee 2013 and b. BDHS 2011 Figure S2: Proportion of meeting complementary feeding criteria by maternal education Figure S3: Proportion of meeting complementary feeding criteria by household wealth Figure S4: Proportion of meeting complementary feeding criteria by community‐level access to health care Figure S5: Proportion of food group consumption in 2007, 2011 and 2014 by survey months Figure S6: Proportion of flesh foods and eggs consumption in 2011 and 2014 [file MCN-14-e12624-s001.pdf]

(A)

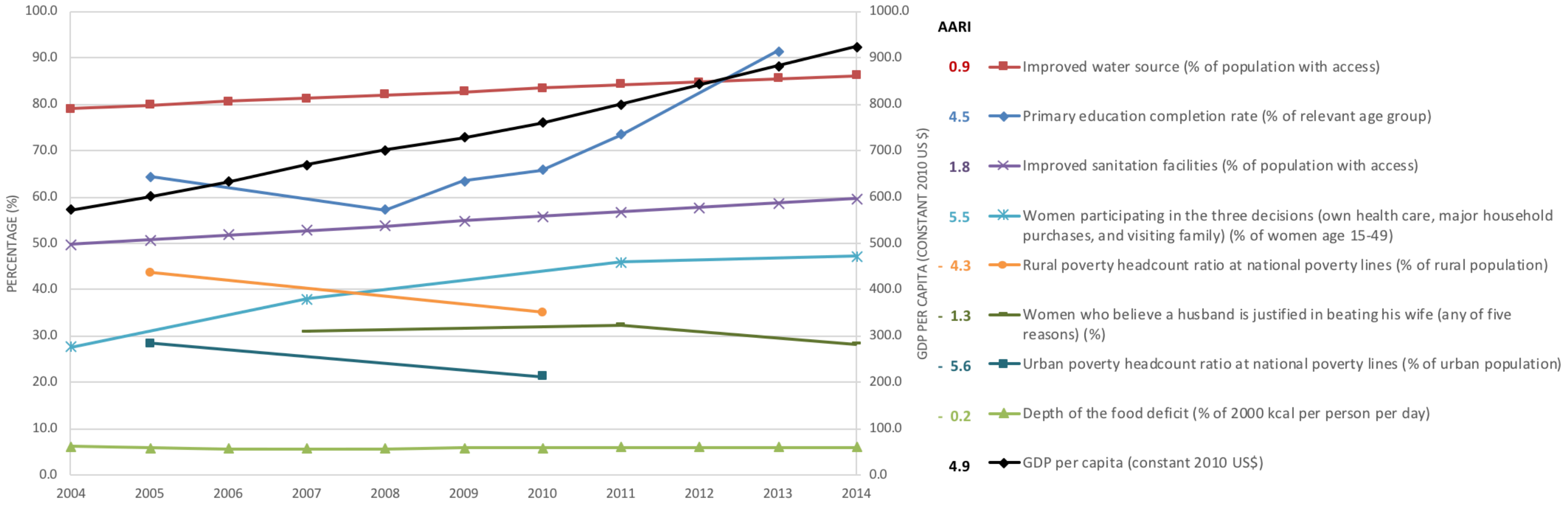
PERCENTAGE (%)
GDP PER CAPITA (CONSTANT 2010 US $)
AARI
0.9 Improved water source (% of population with access)
4.5 Primary education completion rate (% of relevant age group)
1.8 Improved sanitation facilities (% of population with access)
5.5 Women participating in the three decisions (own health care, major household purchases, and visiting family) (% of women age 15-49)
- 4.3 Rural poverty headcount ratio at national poverty lines (% of rural population)
- 1.3 Women who believe a husband is justified in beating his wife (any of five reasons) (%)
- 5.6 Urban poverty headcount ratio at national poverty lines (% of urban population)
- 0.2 Depth of the food deficit (% of 2000 kcal per person per day)
4.9 GDP per capita (constant 2010 US$)
2004 2005 2006 2007 2008 2009 2010 2011 2012 2013 2014

(B)

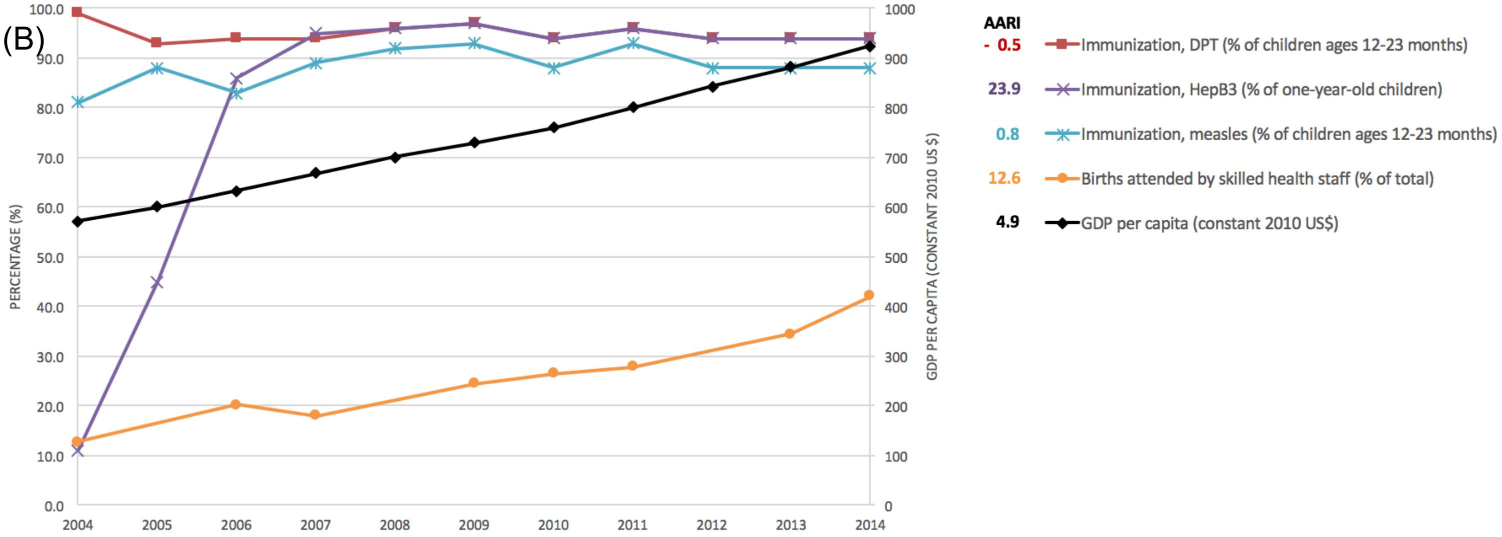
PERCENTAGE (%)
GDP PER CAPITA (CONSTANT 2010 US $)
AARI
- 0.5 Immunization, DPT (% of children ages 12-23 months)
23.9 Immunization, HepB3 (% of one-year-old children)
0.8 Immunization, measles (% of children ages 12-23 months)
12.6 Births attended by skilled health staff (% of total)
4.9 GDP per capita (constant 2010 US$)
2004 2005 2006 2007 2008 2009 2010 2011 2012 2013 2014

(C)

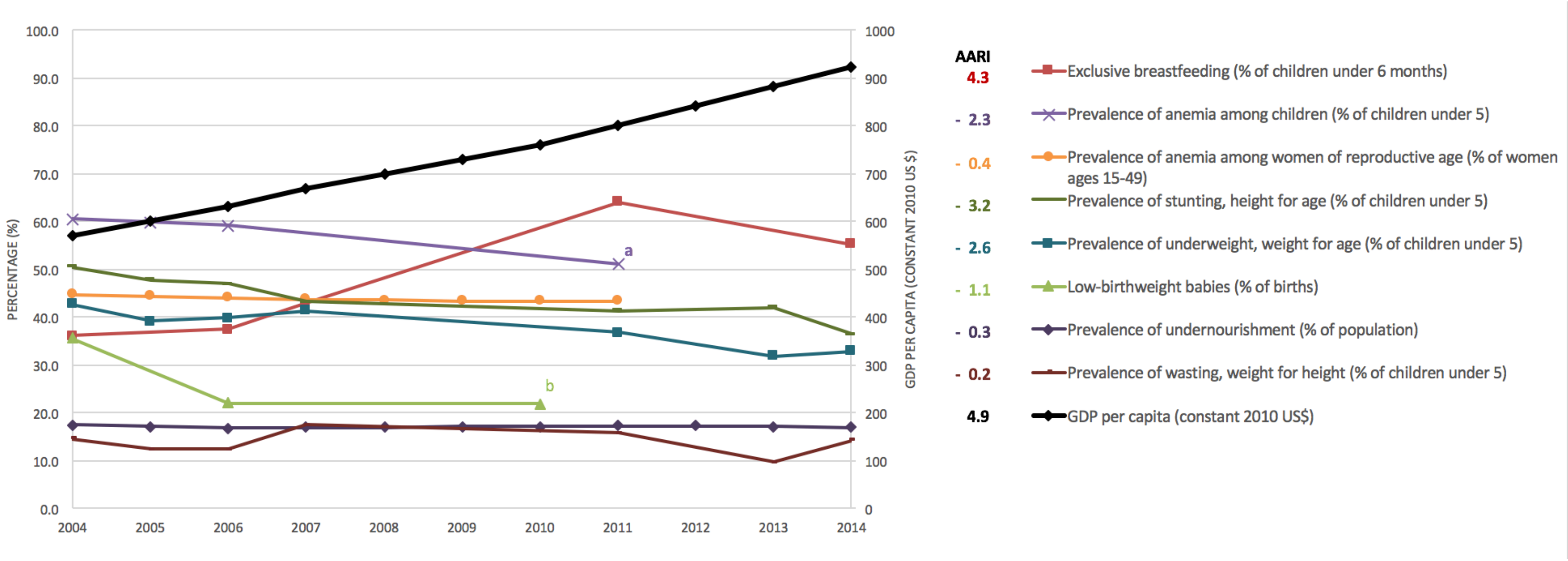


**Supplemental Figure 1:** Changes in (A) socio-economic status; (B) child health indicators; and (C) maternal and child nutritional status between 2004- 2014 in Bangladesh. Data is extracted from World Bank Databank, unless otherwise indicated: a. Lee 2013 and b. BDHS 2011

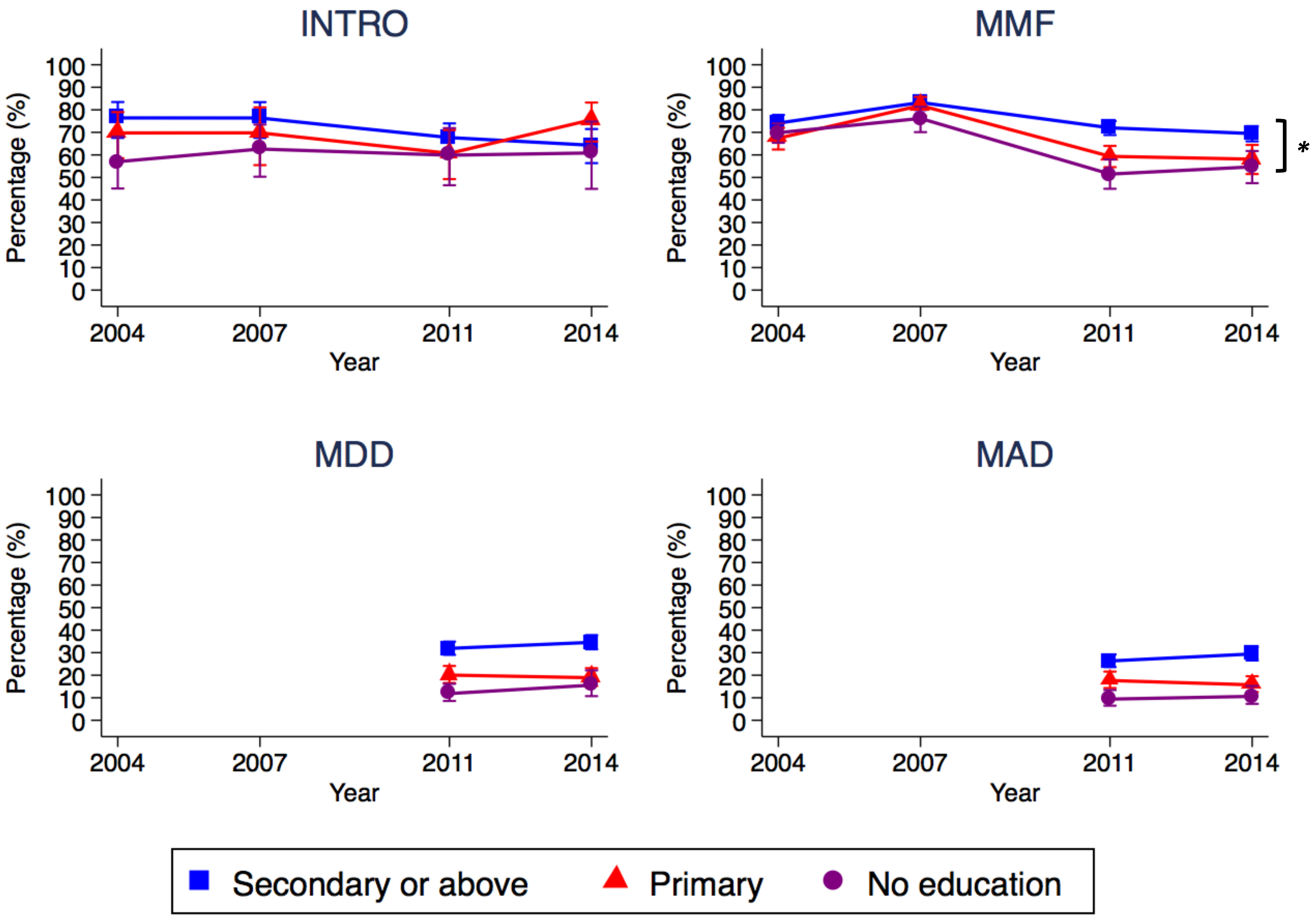


**Supplemental Figure 2**: Proportion of meeting complementary feeding criteria by maternal education

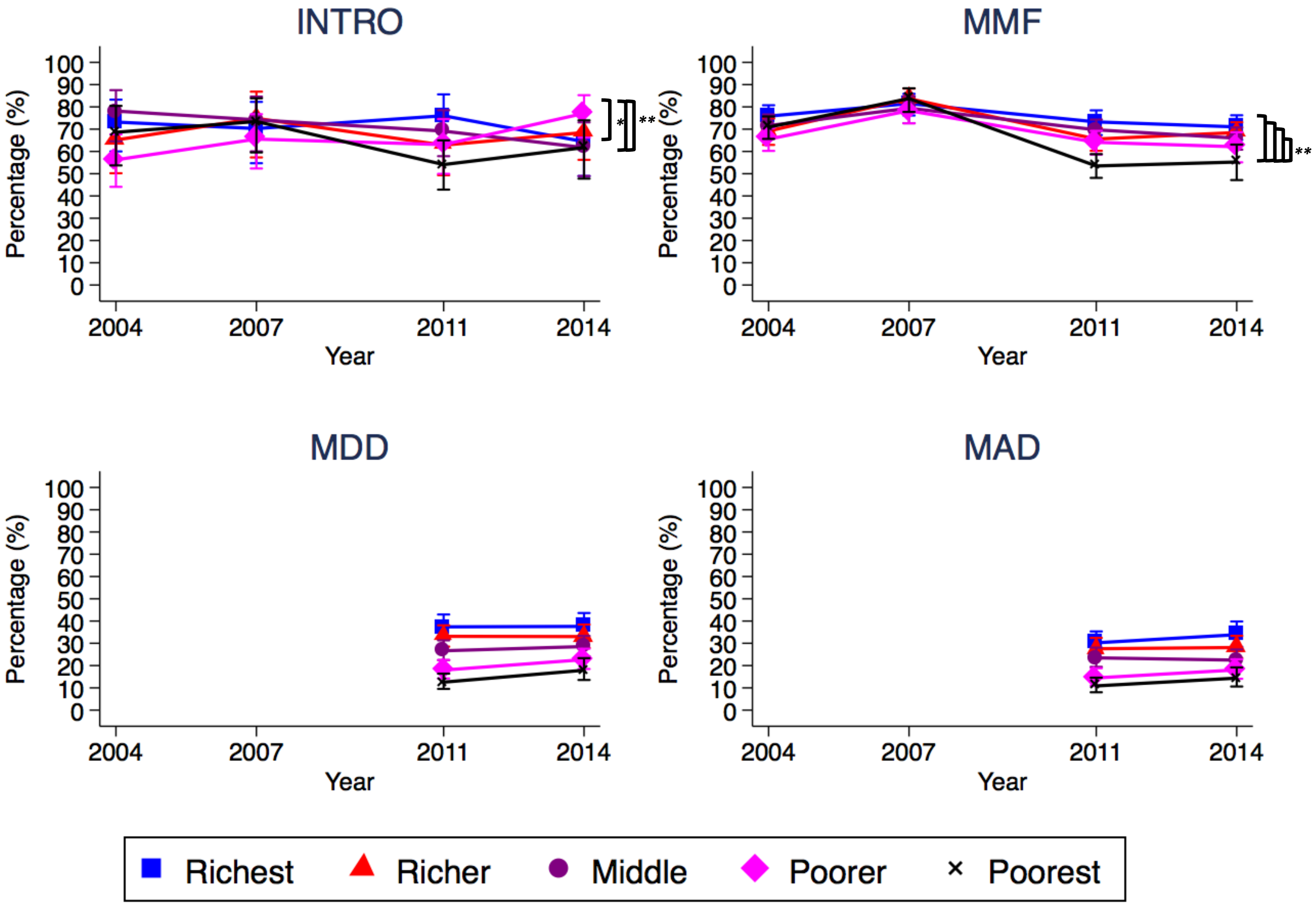


**Supplemental Figure 3**: Proportion of meeting complementary feeding criteria by household wealth

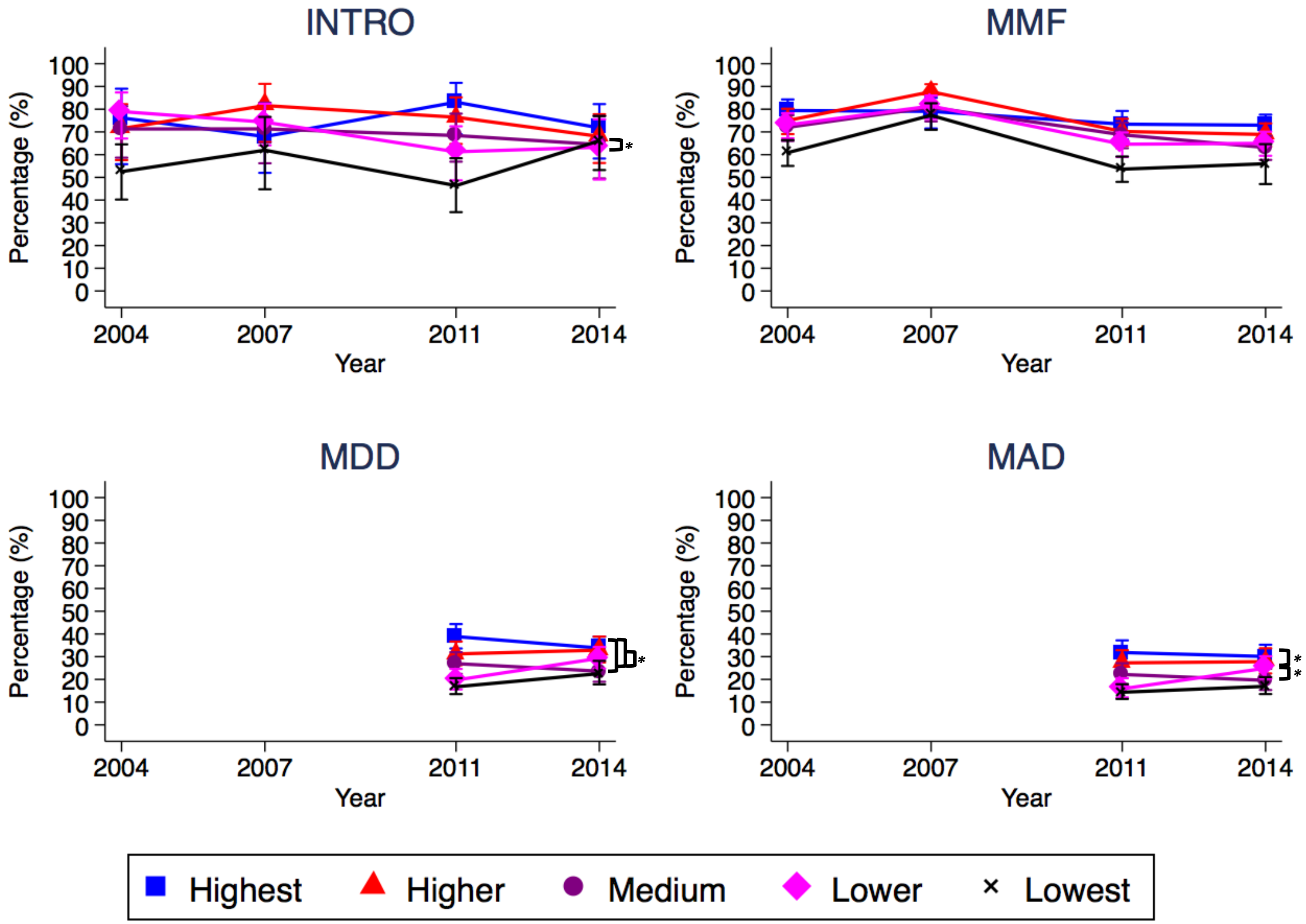


**Supplemental Figure 4**: Proportion of meeting complementary feeding criteria by community-level access to health care

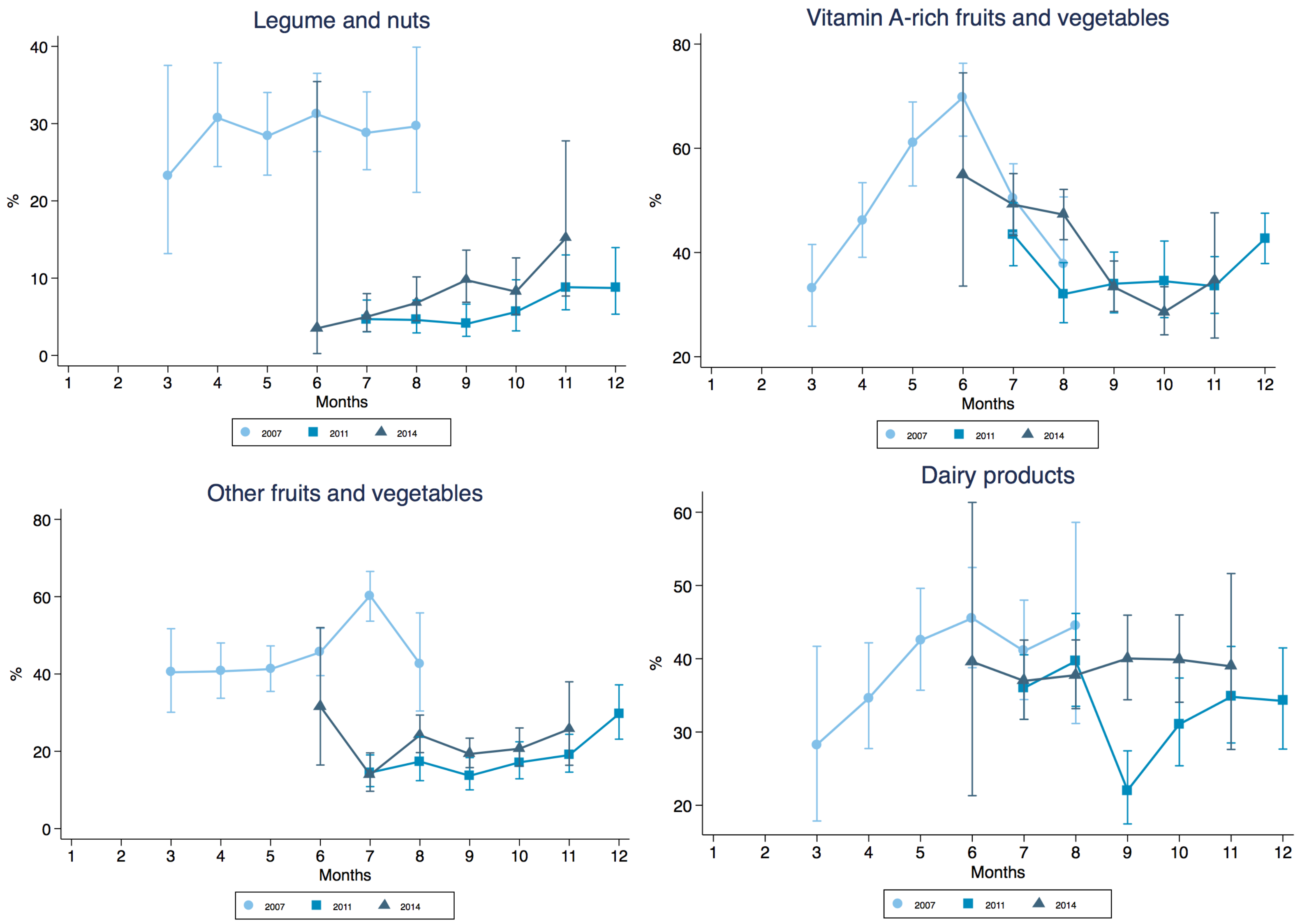


**Supplemental Figure 5**: Proportion of food group consumption in 2007, 2011 and 2014 by survey months

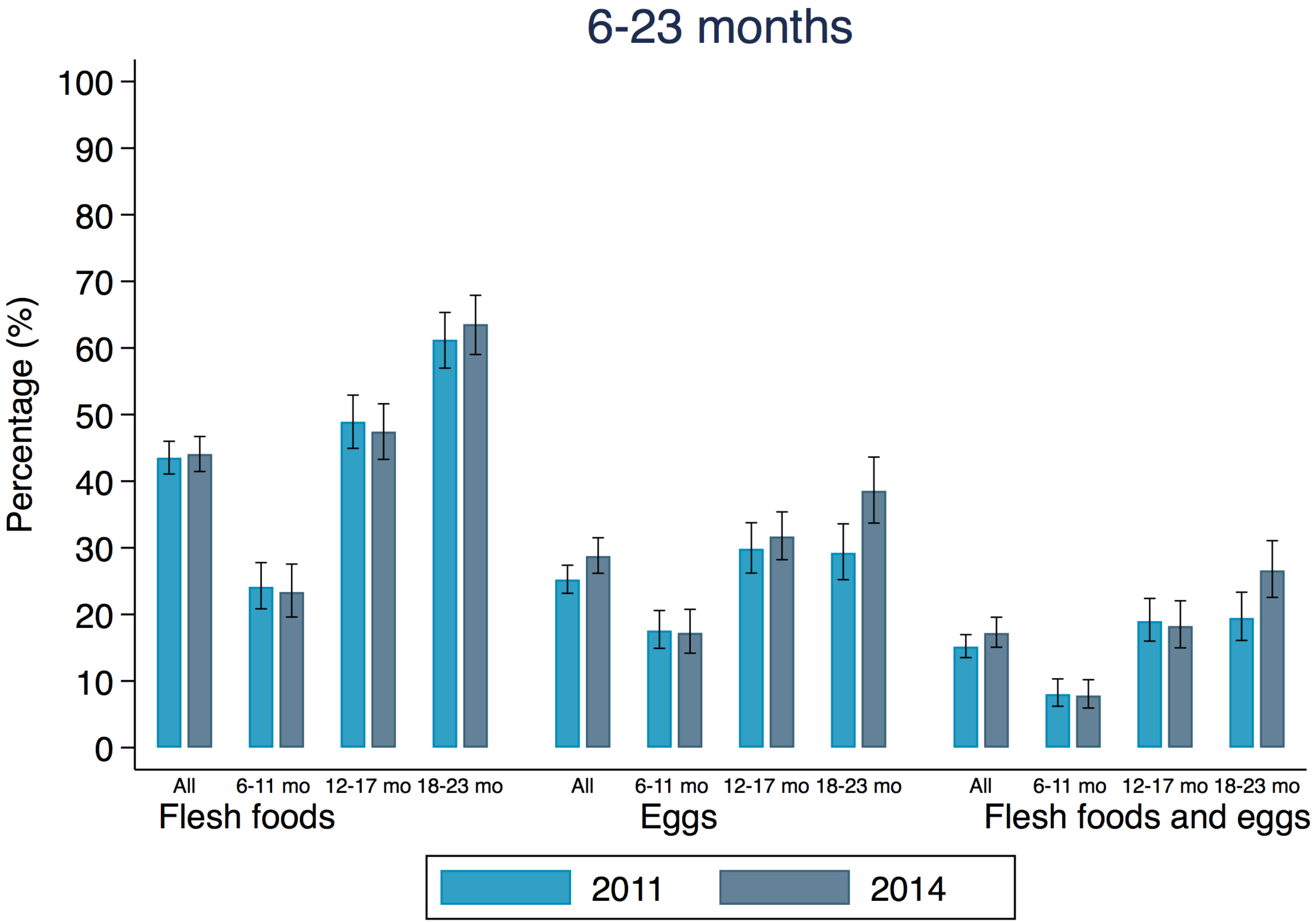


**Supplemental Figure 6**: Proportion of flesh foods and eggs consumption in 2011 and 2014
