# Supplementary material for: Stagnating trends in complementary feeding practices in Bangladesh: An analysis of national surveys from 2004‐2014
Source: Matern Child Nutr. 2018 Jul 12;14(Suppl 4):e12624. doi: 10.1111/mcn.12624 (PMC6586058; doi:10.1111/mcn.12624)
Supplement: Supplementary file 2 — Table S1: Questions about solid, semi‐solid, and soft foods given to the child in the last 24 hours in 2004, 2007, 2011, and 2014 [file MCN-14-e12624-s002.docx]

| **Supplemental Table 1:** Questions about solid, semi-solid, and soft foods given to the child in the last 24 hours in 2004, 2007, 2011, and 2014 | | | | | |
| --- | --- | --- | --- | --- | --- |
| **Question in words** | **2004** | **2007** | **2011** | **2014** |  |
| Bread, noodles, other foods made from grains | N/A | x | x | x |  |
| Baby cereal (commercially fortified cereal) | N/A | N/A | x | x |  |
| Potatoes, cassava or other tubers | N/A | N/A | x | x |  |
| Food make from beans, peas, lentils, nuts | N/A | x | x | x |  |
| Liver heart other organs | N/A | N/A | x | x |  |
| Meat (beef, pork, lamb, chicken, etc) | N/A | x (meat, fish, eggs in one question) | x | x |  |
| Fish or shellfish | N/A |  | x | x |  |
| Eggs | N/A |  | x | x |  |
| Pumpkin, carrots, squash (yellow or orange inside) | N/A | N/A | x | x |  |
| Any DGLV | N/A | x | x | x |  |
| Ripe mangoes, papayas, other vitamin A fruits | N/A | x | x | x |  |
| Any other fruits and vegetables | N/A | x (just other fruits) | x | x (just other fruits) |  |
| Food make from beans, peas, lentils, nuts | N/A | x | x | x |  |
| Powdered or tinned milk | N/A | N/A | x | x |  |
| Baby formula | N/A | x | x | x |  |
| Fresh milk | N/A | x (cow's, goat's milk or yogurt) |  |  |  |
| Yogurt | N/A |  | x | x |  |
| Cheese yogurt, other milk products | N/A | N/A | x | x |  |
| **MDD calculated** |  |  | **x** | **x** |  |
