# Supplementary material for: Stagnating trends in complementary feeding practices in Bangladesh: An analysis of national surveys from 2004‐2014
Source: Matern Child Nutr. 2018 Jul 12;14(Suppl 4):e12624. doi: 10.1111/mcn.12624 (PMC6586058; doi:10.1111/mcn.12624)
Supplement: Supplementary file 3 — Table S2: Factors [OR(95%CI)] in relation to Intro using year‐specific univariate multilevel logistic regression analysis [file MCN-14-e12624-s003.docx]

| **Supplemental Table 2:** Factors [OR(95%CI)] in relation to Intro using year-specific univariate multilevel logistic regression analysis | | | | | | | | | | | | | | | |
| --- | --- | --- | --- | --- | --- | --- | --- | --- | --- | --- | --- | --- | --- | --- | --- |
|  |  |  | 2004 | | | 2007 | | | 2011 | | | 2014 | | |  |
|  |  |  | Estimate | | *P-value* | Estimate | | *P-value* | Estimate | | *P-value* | Estimate | | *P-value* |  |
|  |  |  | OR | (95%CI) |  | OR | (95%CI) |  | OR | (95%CI) |  | OR | (95%CI) |  |  |
| *Child characteristics* | | |  |  |  |  |  |  |  |  |  |  |  |  |  |
|  | Female | | 0.82 | (0.48, 1.40) | *0.46* | 0.56 | (0.33, 0.96) | *** | 0.75 | (0.48, 1.18) | *0.21* | 0.75 | (0.46, 1.20) | *0.23* |  |
|  | Age (months) | |  |  |  |  |  |  |  |  |  |  |  |  |  |
|  |  | 6-11 |  | - |  |  | - |  |  | - |  |  | - |  |  |
|  |  | 12-17 |  | - |  |  | - |  |  | - |  |  | - |  |  |
|  |  | 18-23 |  | - |  |  | - |  |  | - |  |  | - |  |  |
|  | Birth order | |  |  |  |  |  |  |  |  |  |  |  |  |  |
|  |  | Firstborn | 1.06 | (0.60, 1.90) | *0.83* | 0.62 | (0.34, 1.12) | *0.11* | 1.01 | (0.62, 1.65) | *0.95* | 1.47 | (0.89, 2.44) | *0.14* |  |
|  |  | Second to fourth | 1.00 | (Referent) |  | 1.00 | (Referent) |  | 1.00 | (Referent) |  | 1.00 | (Referent) |  |  |
|  |  | Fifth and more | 0.47 | (0.20, 1.07) | *0.07* | 0.65 | (0.28, 1.53) | *0.33* | 0.63 | (0.30, 1.33) | *0.22* | 0.74 | (0.24, 2.35) | *0.61* |  |
|  | Birth interval (month) | |  |  |  |  |  |  |  |  |  |  |  |  |  |
|  |  | No previous birth | 1.17 | (0.65, 2.12) | *0.60* | 0.67 | (0.38, 1.20) | *0.18* | 1.18 | (0.72, 1.92) | *0.52* | 1.49 | (0.89, 2.49) | *0.13* |  |
|  |  | <24 | 0.75 | (0.33, 1.68) | *0.48* | 1.00 | (0.39, 2.59) | *1.00* | 1.97 | (0.74, 5.27) | *0.18* | 0.85 | (0.32, 2.27) | *0.75* |  |
|  |  | >=24 | 1.00 | (Referent) |  | 1.00 | (Referent) |  | 1.00 | (Referent) |  | 1.00 | (Referent) |  |  |
|  | Perceived birth weight | |  |  |  |  |  |  |  |  |  |  |  |  |  |
|  |  | Smaller than average |  | - |  |  | - |  | 0.73 | (0.40, 1.31) | *0.29* | 1.62 | (0.82, 3.17) | *0.16* |  |
|  |  | Average |  | - |  |  | - |  | 1.00 | (Referent) |  | 1.00 | (Referent) |  |  |
|  |  | Larger than average |  | - |  |  | - |  | 1.32 | (0.64, 2.71) | *0.45* | 0.92 | (0.44, 1.94) | *0.83* |  |
|  | Received vitamin A supplementation in the past 6 months | | 1.38 | (0.71, 2.68) | *0.34* | 0.74 | (0.37, 1.50) | *0.41* | 1.87 | (1.07, 3.27) | *** | 1.14 | (0.69, 1.87) | *0.61* |  |
|  | Received iron pills, sprinkles or syrup in the last 7 days | |  | - |  |  | - |  | 4.24 | (0.46, 38.98) | *0.20* | 1.23 | (0.27, 5.67) | *0.79* |  |
|  | Age-appropriate vaccination | |  |  |  |  |  |  |  |  |  |  |  |  |  |
|  |  | None | 0.45 | (0.10, 1.98) | *0.29* | 0.35 | (0.12, 1.03) | *0.06* | 0.57 | (0.20, 1.60) | *0.28* | 0.31 | (0.10, 1.00) | *** |  |
|  |  | Some | 0.48 | (0.25, 0.91) | *** | 0.83 | (0.46, 1.51) | *0.55* | 0.68 | (0.39, 1.19) | *0.18* | 0.59 | (0.32, 1.08) | *0.09* |  |
|  |  | Complete | 1.00 | (Referent) |  | 1.00 | (Referent) |  | 1.00 | (Referent) |  | 1.00 | (Referent) |  |  |
|  | Child health: had the following symptom in the past 2 weeks | |  |  |  |  |  |  |  |  |  |  |  |  |  |
|  |  | Diarrhea | 1.44 | (0.58, 3.63) | *0.43* | 1.44 | (0.61, 3.42) | *0.41* | 1.41 | (0.55, 3.58) | *0.47* | 1.06 | (0.40, 2.86) | *0.90* |  |
|  |  | Fever | 1.14 | (0.66, 1.99) | *0.63* | 1.02 | (0.59, 1.74) | *0.95* | 1.02 | (0.64, 1.60) | *0.94* | 1.05 | (0.65, 1.70) | *0.85* |  |
|  |  | Cough | 1.58 | (0.88, 2.84) | *0.13* | 0.77 | (0.45, 1.33) | *0.35* | 1.33 | (0.85, 2.09) | *0.21* | 0.90 | (0.55, 1.46) | *0.67* |  |
| *Maternal characteristics* | | |  |  |  |  |  |  |  |  |  |  |  |  |  |
|  | Age (years) | |  |  |  |  |  |  |  |  |  |  |  |  |  |
|  |  | 15-24 | 1.32 | (0.71, 2.45) | *0.38* | 0.98 | (0.52, 1.87) | *0.96* | 1.29 | (0.75, 2.23) | *0.36* | 1.15 | (0.65, 2.03) | *0.63* |  |
|  |  | 25-34 | 1.00 | (Referent) |  | 1.00 | (Referent) |  | 1.00 | (Referent) |  | 1.00 | (Referent) |  |  |
|  |  | 35-49 | 0.82 | (0.37, 1.85) | *0.64* | 1.41 | (0.62, 3.22) | *0.41* | 1.14 | (0.55, 2.35) | *0.73* | 1.94 | (0.85, 4.45) | *0.12* |  |
|  | BMI (kg/m^2^) | |  |  |  |  |  |  |  |  |  |  |  |  |  |
|  |  | <18.5 | 0.95 | (0.54, 1.66) | *0.86* | 1.87 | (0.97, 3.60) | *0.06* | 0.85 | (0.52, 1.38) | *0.51* | 1.15 | (0.65, 2.06) | *0.63* |  |
|  |  | 18.5-24.9 | 1.00 | (Referent) |  | 1.00 | (Referent) |  | 1.00 | (Referent) |  | 1.00 | (Referent) |  |  |
|  |  | >=25 | 0.90 | (0.22, 3.59) | *0.88* | 0.41 | (0.12, 1.43) | *0.16* | 1.09 | (0.48, 2.48) | *0.83* | 0.85 | (0.40, 1.80) | *0.67* |  |
|  | Reproductive health care | |  |  |  |  |  |  |  |  |  |  |  |  |  |
|  |  | Delivered at health facility | 1.79 | (0.81, 3.95) | *0.15* | 0.56 | (0.29, 1.10) | *0.09* | 2.12 | (1.24, 3.62) | **** | 1.01 | (0.62, 1.66) | *0.97* |  |
|  |  | Type of delivery assistance |  |  |  |  |  |  |  |  |  |  |  |  |  |
|  |  | Health professional | 1.42 | (0.71, 2.84) | *0.33* | 0.64 | (0.34, 1.22) | *0.18* | 2.00 | (1.18, 3.39) | *** | 0.96 | (0.58, 1.60) | *0.87* |  |
|  |  | Traditional birth attendant | 0.73 | (0.31, 1.69) | *0.46* | 1.68 | (0.68, 4.18) | *0.26* | 1.62 | (0.77, 3.40) | *0.20* | 0.87 | (0.36, 2.11) | *0.76* |  |
|  |  | Other | 1.00 | (Referent) |  | 1.00 | (Referent) |  | 1.00 | (Referent) |  | 1.00 | (Referent) |  |  |
|  |  | Caesarean delivery | 1.68 | (0.46, 6.09) | *0.43* | 0.48 | (0.19, 1.20) | *0.11* | 2.57 | (1.25, 5.28) | *** | 0.94 | (0.53, 1.68) | *0.83* |  |
|  | | | | | | | | | | | | | | | |
| **Supplemental Table 2 cont’** | | | | | | | | | | | | | | | |
|  |  |  | 2004 | | | 2007 | | | 2011 | | | 2014 | | |  |
|  |  |  | Estimate | | *P-value* | Estimate | | *P-value* | Estimate | | *P-value* | Estimate | | *P-value* |  |
|  |  |  | OR | (95%CI) |  | OR | (95%CI) |  | OR | (95%CI) |  | OR | (95%CI) |  |  |
|  |  | Antenatal clinic visits |  |  |  |  |  |  |  |  |  |  |  |  |  |
|  |  | None | 0.31 | (0.16, 0.61) | **** | 0.87 | (0.48, 1.59) | *0.65* | 0.54 | (0.33, 0.90) | *** | 0.66 | (0.34, 1.31) | *0.24* |  |
|  |  | 1-3 | 1.00 | (Referent) |  | 1.00 | (Referent) |  | 1.00 | (Referent) |  | 1.00 | (Referent) |  |  |
|  |  | ≥4 | 0.88 | (0.41, 1.92) | *0.75* | 1.00 | (0.48, 2.09) | *0.99* | 1.39 | (0.80, 2.44) | *0.24* | 1.43 | (0.82, 2.50) | *0.21* |  |
|  |  | Postnatal check-up on woman |  |  |  |  |  |  |  |  |  |  |  |  |  |
|  |  | 0-1d | 1.00 | (Referent) |  | 1.00 | (Referent) |  | 1.00 | (Referent) |  | 1.00 | (Referent) |  |  |
|  |  | >=2d | 1.15 | (0.29, 4.62) | *0.84* | 0.23 | (0.06, 0.90) | *** | 0.72 | (0.22, 2.32) | *0.58* | 0.53 | (0.20, 1.37) | *0.19* |  |
|  |  | Missing or unknown | 0.96 | (0.38, 2.42) | *0.93* | 1.08 | (0.61, 1.94) | *0.79* | 0.50 | (0.29, 0.88) | *** | 0.96 | (0.56, 1.65) | *0.89* |  |
|  |  | Postnatal check-up on child |  |  |  |  |  |  |  |  |  |  |  |  |  |
|  |  | 0-1d |  | - |  | 1.00 | (Referent) |  | 1.00 | (Referent) |  | 1.00 | (Referent) |  |  |
|  |  | >=2d |  | - |  | 1.37 | (0.40, 4.77) | *0.62* | 1.60 | (0.77, 3.34) | *0.21* | 1.37 | (0.61, 3.08) | *0.44* |  |
|  |  | Missing or unknown |  | - |  | 1.12 | (0.61, 2.05) | *0.71* | 0.68 | (0.42, 1.10) | *0.12* | 1.00 | (0.59, 1.72) | *0.99* |  |
|  | Maternal education | |  |  |  |  |  |  |  |  |  |  |  |  |  |
|  |  | No education | 0.36 | (0.19, 0.69) | **** | 0.43 | (0.21, 0.85) | *** | 0.67 | (0.36, 1.26) | *0.21* | 0.87 | (0.42, 1.79) | *0.71* |  |
|  |  | Primary | 0.78 | (0.40, 1.53) | *0.47* | 0.56 | (0.29, 1.08) | *0.08* | 0.66 | (0.40, 1.11) | *0.12* | 1.35 | (0.77, 2.39) | *0.30* |  |
|  |  | Secondary or higher | 1.00 | (Referent) |  | 1.00 | (Referent) |  | 1.00 | (Referent) |  | 1.00 | (Referent) |  |  |
|  | Exposure to media: at least once a week | |  |  |  |  |  |  |  |  |  |  |  |  |  |
|  |  | Reading newspaper | 1.33 | (0.51, 3.49) | *0.56* | 0.57 | (0.21, 1.58) | *0.28* | 0.81 | (0.27, 2.40) | *0.70* | 0.53 | (0.16, 1.80) | *0.31* |  |
|  |  | Listening to radio | 1.17 | (0.66, 2.06) | *0.60* | 1.09 | (0.54, 2.19) | *0.81* | 1.69 | (0.65, 4.39) | *0.29* | 0.79 | (0.19, 3.29) | *0.74* |  |
|  |  | Watching TV | 2.01 | (1.12, 3.61) | *** | 1.02 | (0.59, 1.78) | *0.93* | 1.66 | (1.05, 2.62) | *** | 1.37 | (0.83, 2.25) | *0.22* |  |
|  | Involved in decision making on | |  |  |  |  |  |  |  |  |  |  |  |  |  |
|  |  | How man's income is used |  | - |  |  | - |  |  | - |  |  | - |  |  |
|  |  | Large household purchases | 1.20 | (0.70, 2.05) | *0.50* | 1.43 | (0.84, 2.43) | *0.18* | 1.20 | (0.77, 1.88) | *0.42* | 1.68 | (1.03, 2.73) | *** |  |
|  |  | Visiting family and friends | 1.06 | (0.59, 1.90) | *0.84* | 1.51 | (0.90, 2.53) | *0.12* | 0.85 | (0.54, 1.32) | *0.47* | 1.67 | (1.01, 2.77) | *** |  |
|  |  | Regarding own health care | 1.15 | (0.65, 2.04) | *0.64* | 1.22 | (0.72, 2.08) | *0.46* | 0.81 | (0.52, 1.26) | *0.35* | 1.79 | (1.08, 2.95) | *** |  |
|  | Appropriate attitude towards domestic violence: no queried situation was justified | |  | - |  | 0.78 | (0.42, 1.43) | *0.42* | 0.75 | (0.46, 1.22) | *0.25* | 1.28 | (0.75, 2.20) | *0.37* |  |
|  | Women's empowerment score (5 items) | |  |  |  |  |  |  |  |  |  |  |  |  |  |
|  |  | <Weighted mean | 1.00 | (Referent) |  | 1.00 | (Referent) |  | 1.00 | (Referent) |  | 1.00 | (Referent) |  |  |
|  |  | >=Weighted mean | 1.54 | (0.85, 2.81) | *0.16* | 1.21 | (0.71, 2.05) | *0.48* | 0.91 | (0.58, 1.43) | *0.69* | 1.89 | (1.13, 3.14) | *** |  |
| *Paternal characteristics* | | |  |  |  |  |  |  |  |  |  |  |  |  |  |
|  | Age (years) | |  |  |  |  |  |  |  |  |  |  |  |  |  |
|  |  | < 31 | 1.00 | (Referent) |  | 1.00 | (Referent) |  | 1.00 | (Referent) |  | 1.00 | (Referent) |  |  |
|  |  | >=31 | 0.50 | (0.29, 0.88) | *** | 0.82 | (0.48, 1.40) | *0.47* | 1.17 | (0.75, 1.82) | *0.49* | 1.03 | (0.63, 1.69) | *0.89* |  |
|  | Highest educational level | |  |  |  |  |  |  |  |  |  |  |  |  |  |
|  |  | No education | 0.43 | (0.22, 0.83) | *** | 0.91 | (0.49, 1.70) | *0.77* | 0.54 | (0.31, 0.92) | *** | 0.65 | (0.34, 1.26) | *0.20* |  |
|  |  | Primary | 0.58 | (0.29, 1.17) | *0.13* | 1.15 | (0.58, 2.30) | *0.69* | 1.00 | (0.58, 1.71) | *0.99* | 1.81 | (1.00, 3.29) | *0.05* |  |
|  |  | Secondary or higher | 1.00 | (Referent) |  | 1.00 | (Referent) |  | 1.00 | (Referent) |  | 1.00 | (Referent) |  |  |
| *Household characteristics* | | |  |  |  |  |  |  |  |  |  |  |  |  |  |
|  | Female household head | | 0.26 | (0.08, 0.88) | *** | 0.81 | (0.36, 1.81) | *0.60* | 0.79 | (0.33, 1.91) | *0.60* | 1.41 | (0.55, 3.64) | *0.47* |  |
|  | No. of HH members | |  |  |  |  |  |  |  |  |  |  |  |  |  |
|  |  | <Weighted mean (9.0) | 1.00 | (Referent) |  | 1.00 | (Referent) |  | 1.00 | (Referent) |  | 1.00 | (Referent) |  |  |
|  |  | >=Weighted mean (9.0) | 0.78 | (0.45, 1.35) | **** | 0.89 | (0.52, 1.54) | **** | 0.87 | (0.55, 1.38) | *0.54* | 1.11 | (0.67, 1.86) | *0.68* |  |
|  | | | | | | | | | | | | | | | |
| **Supplemental Table 2 cont’** | | | | | | | | | | | | | | | |
|  |  |  | 2004 | | | 2007 | | | 2011 | | | 2014 | | |  |
|  |  |  | Estimate | | *P-value* | Estimate | | *P-value* | Estimate | | *P-value* | Estimate | | *P-value* |  |
|  |  |  | OR | (95%CI) |  | OR | (95%CI) |  | OR | (95%CI) |  | OR | (95%CI) |  |  |
|  | No. of children under 5 years | |  |  |  |  |  |  |  |  |  |  |  |  |  |
|  |  | <Weighted mean (2.3) | 1.00 | (Referent) |  | 1.00 | (Referent) |  | 1.00 | (Referent) |  | 1.00 | (Referent) |  |  |
|  |  | >=Weighted mean (2.3) | 0.61 | (0.35, 1.06) | *0.08* | 1.08 | (0.63, 1.85) | *0.78* | 0.72 | (0.45, 1.15) | *0.16* | 0.73 | (0.44, 1.19) | *0.20* |  |
|  | Type of cooking fuel | |  |  |  |  |  |  |  |  |  |  |  |  |  |
|  |  | Electricity, LPG, natural gas, biogas | 2.15 | (0.72, 6.44) | *0.17* | 0.45 | (0.16, 1.24) | *0.12* | 1.16 | (0.49, 2.79) | *0.74* | 0.72 | (0.35, 1.47) | *0.36* |  |
|  |  | Wood, straw/ shrubs/ grass, animal dung and other | 1.00 | (Referent) |  | 1.00 | (Referent) |  | 1.00 | (Referent) |  | 1.00 | (Referent) |  |  |
|  | Water source | |  |  |  |  |  |  |  |  |  |  |  |  |  |
|  |  | Unimproved source of drinking water | 0.45 | (0.12, 1.61) | *0.22* | 2.08 | (0.49, 8.83) | *0.32* | 1.69 | (0.29, 9.78) | *0.56* | 2.65 | (0.46, 15.27) | *0.28* |  |
|  |  | Source for water not in own dwelling or yard/plot |  | - |  |  | - |  | 0.72 | (0.44, 1.19) | *0.20* | 0.79 | (0.47, 1.34) | *0.38* |  |
|  |  | Time to get to water source  (min) |  |  |  |  |  |  |  |  |  |  |  |  |  |
|  |  | 0 |  | - |  |  | - |  | 1.00 | (Referent) | *0* | 1.00 | (Referent) | *0* |  |
|  |  | 1-59 |  | - |  |  | - |  | 0.66 | (0.41, 1.06) | *0.09* | 0.79 | (0.46, 1.37) | *0.41* |  |
|  |  | >=60 |  | - |  |  | - |  |  | - | *0.99* | 1.43 | (0.11, 19.18) | *0.79* |  |
|  | Toilet condition | |  |  |  |  |  |  |  |  |  |  |  |  |  |
|  |  | Unimproved toilet facility | 0.90 | (0.51, 1.60) | *0.72* | 0.80 | (0.46, 1.40) | *0.44* | 0.63 | (0.40, 0.99) | *** | 0.69 | (0.40, 1.19) | *0.18* |  |
|  |  | Shared toilet with other households |  |  |  | 1.63 | (0.89, 2.99) | *0.11* | 0.64 | (0.39, 1.07) | *0.09* | 1.63 | (0.96, 2.75) | *0.07* |  |
|  | HH wealth | |  |  |  |  |  |  |  |  |  |  |  |  |  |
|  |  | Richest | 1.00 | (Referent) |  | 1.00 | (Referent) |  | 1.00 | (Referent) |  | 1.00 | (Referent) |  |  |
|  |  | Richer | 0.62 | (0.27, 1.42) | *0.26* | 1.08 | (0.44, 2.63) | *0.86* | 0.59 | (0.27, 1.29) | *0.19* | 1.11 | (0.50, 2.44) | *0.80* |  |
|  |  | Middle | 1.09 | (0.45, 2.61) | *0.85* | 1.24 | (0.52, 2.97) | *0.63* | 0.88 | (0.40, 1.94) | *0.74* | 0.81 | (0.37, 1.77) | *0.60* |  |
|  |  | Poorer | 0.46 | (0.21, 1.02) | *0.06* | 0.76 | (0.34, 1.71) | *0.51* | 0.59 | (0.26, 1.30) | *0.19* | 1.32 | (0.59, 2.94) | *0.50* |  |
|  |  | Poorest | 0.76 | (0.34, 1.69) | *0.50* | 1.11 | (0.45, 2.75) | *0.82* | 0.34 | (0.16, 0.73) | **** | 0.84 | (0.38, 1.84) | *0.66* |  |
| *Community characteristics* | | |  |  |  |  |  |  |  |  |  |  |  |  |  |
|  | Rural residence | | 0.91 | (0.49, 1.68) | *0.76* | 0.90 | (0.49, 1.64) | *0.73* | 0.72 | (0.42, 1.21) | *0.21* | 0.84 | (0.49, 1.44) | *0.53* |  |
|  | Geographical region | |  |  |  |  |  |  |  |  |  |  |  |  |  |
|  |  | Barisal | 1.00 | (Referent) |  | 1.00 | (Referent) |  | 1.00 | (Referent) |  | 1.00 | (Referent) |  |  |
|  |  | Chittagong | 0.77 | (0.28, 2.11) | *0.62* | 0.67 | (0.26, 1.71) | *0.40* | 0.72 | (0.33, 1.56) | *0.41* | 0.57 | (0.22, 1.43) | *0.23* |  |
|  |  | Dhaka | 1.11 | (0.40, 3.08) | *0.84* | 1.51 | (0.59, 3.85) | *0.38* | 1.96 | (0.83, 4.64) | *0.12* | 0.85 | (0.33, 2.18) | *0.73* |  |
|  |  | Khulna | 1.25 | (0.38, 4.10) | *0.71* | 2.87 | (0.87, 9.41) | *0.08* | 2.94 | (0.98, 8.79) | *0.05* | 1.12 | (0.39, 3.23) | *0.84* |  |
|  |  | Rajshahi | 2.04 | (0.66, 6.29) | *0.22* | 6.57 | (1.81, 23.85) | **** | 3.45 | (1.48, 8.06) | **** | 0.94 | (0.38, 2.29) | *0.89* |  |
|  |  | Sylhet | 0.62 | (0.19, 1.98) | *0.42* | 1.18 | (0.44, 3.19) | *0.74* | 1.11 | (0.48, 2.55) | *0.81* | 0.59 | (0.23, 1.55) | *0.29* |  |
|  | Women completed primary or higher education | | 4.79 | (1.12, 20.40) | *0.73* | 1.48 | (0.31, 7.07) | *0.62* | 2.04 | (28.04, 0.00) | **** | 0.90 | (26.22, 0.07) | *0.15* |  |
|  | Women's empowerment | | 1.60 | (0.83, 3.07) | *0.86* | 2.06 | (1.20, 3.55) | **** | 0.90 | (2.20, 0.14) | *** | 0.65 | (1.75, 0.81) | *0.17* |  |
|  | Rank of access to health care | |  |  |  |  |  |  |  |  |  |  |  |  |  |
|  |  | Highest (best access) | 1.00 | (Referent) |  | 1.00 | (Referent) |  | 1.00 | (Referent) |  | 1.00 | (Referent) |  |  |
|  |  | Higher | 0.57 | (0.22, 1.50) | *0.26* | 1.91 | (0.66, 5.56) | *0.24* | 0.64 | (0.27, 1.52) | *0.31* | 0.69 | (0.31, 1.56) | *0.37* |  |
|  |  | Medium | 0.51 | (0.20, 1.30) | *0.16* | 1.39 | (0.51, 3.75) | *0.52* | 0.46 | (0.20, 1.03) | *0.06* | 0.82 | (0.35, 1.90) | *0.64* |  |
|  |  | Lower | 0.95 | (0.36, 2.55) | *0.93* | 1.43 | (0.56, 3.66) | *0.45* | 0.33 | (0.15, 0.74) | **** | 0.62 | (0.27, 1.41) | *0.25* |  |
|  |  | Lowest (worse access) | 0.29 | (0.12, 0.72) | **** | 0.84 | (0.34, 2.07) | *0.70* | 0.17 | (0.08, 0.38) | **** | 0.74 | (0.33, 1.65) | *0.45* |  |
|  | Unimproved toilet | | 0.40 | (0.13, 1.18) | *0.86* | 0.59 | (0.20, 1.76) | *0.34* | 0.11 | (0.66, 0.00) | **** | 0.15 | (1.15, 0.09) | *0.16* |  |
|  | Share toilet with other households | |  | - |  | 1.76 | (0.41, 7.51) | *0.44* | 0.14 | (1.64, 0.24) | *** | 0.18 | (2.43, 0.53) | *0.17* |  |
