# Supplementary material for: Stagnating trends in complementary feeding practices in Bangladesh: An analysis of national surveys from 2004‐2014
Source: Matern Child Nutr. 2018 Jul 12;14(Suppl 4):e12624. doi: 10.1111/mcn.12624 (PMC6586058; doi:10.1111/mcn.12624)
Supplement: Supplementary file 4 — Table S3: Factors [OR(95%CI)] in relation to MMF using year‐specific univariate multilevel logistic regression analysis [file MCN-14-e12624-s004.docx]

| **Supplemental Table 3:** Factors [OR(95%CI)] in relation to MMF using year-specific univariate multilevel logistic regression analysis | | | | | | | | | | | | | | | | | | | | | | | | | | | | | | | | | | | | | | | | | | | | |  | | | | | | |
| --- | --- | --- | --- | --- | --- | --- | --- | --- | --- | --- | --- | --- | --- | --- | --- | --- | --- | --- | --- | --- | --- | --- | --- | --- | --- | --- | --- | --- | --- | --- | --- | --- | --- | --- | --- | --- | --- | --- | --- | --- | --- | --- | --- | --- | --- | --- | --- | --- | --- | --- | --- |
|  | | |  | | |  | | | 2004 | | | | | | | | | | 2007 | | | | | | | | | | 2011 | | | | | | | | | | 2014 | | | | | | | | | | | | |
|  | | |  | | |  | | | Estimate | | | | | | *P-value* | | | | Estimate | | | | | | *P-value* | | | | Estimate | | | | | | *P-value* | | | | Estimate | | | | | | *P-value* | | | | | |  |
|  | | |  | | |  | | | OR | | | (95%CI) | | |  |  |  |  | OR | | | (95%CI) | | |  |  |  |  | OR | | | (95%CI) | | |  |  |  |  | OR | | | (95%CI) | | |  |  |  |  |  |  |  |
| *Child characteristics* | | | | | | | | |  | | |  | | |  | | | |  | | |  | | |  | | | |  | | |  | | |  | | | |  | | |  | | |  | | | | | |  |
|  | | | Female | | | | | | 1.12 | | | (0.96, 1.30) | | | *0.15* | | | | 0.98 | | | (0.84, 1.14) | | | *0.82* | | | | 0.94 | | | (0.78, 1.14) | | | *0.54* | | | | 0.92 | | | (0.76, 1.10) | | | *0.34* | | | | | |  |
|  | | | Age (months) | | | | | |  | | |  | | |  | | | |  | | |  | | |  | | | |  | | |  | | |  | | | |  | | |  | | |  | | | | | |  |
|  | | |  | | | 6-11 | | | 1.00 | | | (Referent) | | |  | | | | 1.00 | | | (Referent) | | |  | | | | 1.00 | | | (Referent) | | |  | | | | 1.00 | | | (Referent) | | |  | | | | | |  |
|  | | |  | | | 12-17 | | | 1.98 | | | (1.54, 2.55) | | | **** | | | | 3.17 | | | (2.30, 4.36) | | | **** | | | | 1.72 | | | (1.37, 2.15) | | | **** | | | | 2.21 | | | (1.77, 2.77) | | | **** | | | | | |  |
|  | | |  | | | 18-23 | | | 4.59 | | | (3.33, 6.33) | | | **** | | | | 8.40 | | | (5.58, 12.63) | | | **** | | | | 2.63 | | | (2.06, 3.35) | | | **** | | | | 2.69 | | | (2.12, 3.40) | | | **** | | | | | |  |
|  | | | Birth order | | | | | |  | | |  | | |  | | | |  | | |  | | |  | | | |  | | |  | | |  | | | |  | | |  | | |  | | | | | |  |
|  | | |  | | | Firstborn | | | 1.35 | | | (1.14, 1.60) | | | **** | | | | 1.30 | | | (1.10, 1.54) | | | **** | | | | 1.29 | | | (1.05, 1.57) | | | *** | | | | 0.99 | | | (0.82, 1.20) | | | *0.94* | | | | | |  |
|  | | |  | | | Second to fourth | | | 1.00 | | | (Referent) | | |  | | | | 1.00 | | | (Referent) | | |  | | | | 1.00 | | | (Referent) | | |  | | | | 1.00 | | | (Referent) | | |  | | | | | |  |
|  | | |  | | | Fifth and more | | | 0.79 | | | (0.64, 0.99) | | | *** | | | | 0.79 | | | (0.62, 1.00) | | | *** | | | | 0.82 | | | (0.59, 1.16) | | | *0.26* | | | | 0.78 | | | (0.53, 1.14) | | | *0.20* | | | | | |  |
|  | | | Birth interval (month) | | | | | |  | | |  | | |  | | | |  | | |  | | |  | | | |  | | |  | | |  | | | |  | | |  | | |  | | | | | |  |
|  | | |  | | | No previous birth | | | 1.42 | | | (1.20, 1.68) | | | **** | | | | 1.36 | | | (1.15, 1.61) | | | **** | | | | 1.46 | | | (1.19, 1.79) | | | **** | | | | 1.03 | | | (0.85, 1.25) | | | *0.77* | | | | | |  |
|  | | |  | | | <24 | | | 1.00 | | | (0.78, 1.28) | | | *0.97* | | | | 0.98 | | | (0.75, 1.27) | | | *0.86* | | | | 2.14 | | | (1.46, 3.14) | | | **** | | | | 1.10 | | | (0.74, 1.63) | | | *0.65* | | | | | |  |
|  | | |  | | | >=24 | | | 1.00 | | | (Referent) | | |  | | | | 1.00 | | | (Referent) | | |  | | | | 1.00 | | | (Referent) | | |  | | | | 1.00 | | | (Referent) | | |  | | | | | |  |
|  | | | Perceived birth weight | | | | | |  | | |  | | |  | | | |  | | |  | | |  | | | |  | | |  | | |  | | | |  | | |  | | |  | | | | | |  |
|  | | |  | | | Smaller than average | | |  | | | - | | |  | | | |  | | | - | | |  | | | | 0.89 | | | (0.70, 1.14) | | | *0.35* | | | | 0.86 | | | (0.68, 1.09) | | | *0.21* | | | | | |  |
|  | | |  | | | Average | | |  | | | - | | |  | | | |  | | | - | | |  | | | | 1.00 | | | (Referent) | | |  | | | | 1.00 | | | (Referent) | | |  | | | | | |  |
|  | | |  | | | Larger than average | | |  | | | - | | |  | | | |  | | | - | | |  | | | | 1.14 | | | (0.87, 1.51) | | | *0.35* | | | | 1.10 | | | (0.83, 1.47) | | | *0.49* | | | | | |  |
|  | | | Received vitamin A supplementation in the past 6 months | | | | | | 0.55 | | | (0.46, 0.66) | | | **** | | | | 0.51 | | | (0.42, 0.61) | | | **** | | | | 1.38 | | | (1.13, 1.67) | | | **** | | | | 1.57 | | | (1.30, 1.89) | | | **** | | | | | |  |
|  | | | Received iron pills, sprinkles or syrup in the last 7 days | | | | | |  | | | - | | |  | | | |  | | | - | | |  | | | | 1.35 | | | (0.73, 2.48) | | | *0.34* | | | | 0.89 | | | (0.59, 1.36) | | | *0.60* | | | | | |  |
|  | | | Age-appropriate vaccination | | | | | |  | | |  | | |  | | | |  | | |  | | |  | | | |  | | |  | | |  | | | |  | | |  | | |  | | | | | |  |
|  | | |  | | | None | | | 1.17 | | | (0.73, 1.88) | | | *0.50* | | | | 0.97 | | | (0.60, 1.55) | | | *0.89* | | | | 0.48 | | | (0.27, 0.87) | | | *** | | | | 0.57 | | | (0.33, 0.97) | | | *** | | | | | |  |
|  | | |  | | | Some | | | 1.04 | | | (0.87, 1.25) | | | *0.67* | | | | 1.23 | | | (1.00, 1.52) | | | *0.05* | | | | 0.64 | | | (0.50, 0.83) | | | **** | | | | 0.62 | | | (0.49, 0.78) | | | **** | | | | | |  |
|  | | |  | | | Complete | | | 1.00 | | | (Referent) | | |  | | | | 1.00 | | | (Referent) | | |  | | | | 1.00 | | | (Referent) | | |  | | | | 1.00 | | | (Referent) | | |  | | | | | |  |
|  | | | Child health: had the following symptom in the past 2 weeks | | | | | |  | | |  | | |  | | | |  | | |  | | |  | | | |  | | |  | | |  | | | |  | | |  | | |  | | | | | |  |
|  | | |  | | | Diarrhea | | | 1.34 | | | (1.05, 1.72) | | | *** | | | | 1.21 | | | (0.96, 1.52) | | | *0.11* | | | | 0.91 | | | (0.64, 1.29) | | | *0.61* | | | | 0.99 | | | (0.70, 1.40) | | | *0.94* | | | | | |  |
|  | | |  | | | Fever | | | 1.22 | | | (1.05, 1.42) | | | *** | | | | 1.09 | | | (0.93, 1.27) | | | *0.29* | | | | 0.91 | | | (0.75, 1.10) | | | *0.33* | | | | 0.83 | | | (0.69, 1.00) | | | *** | | | | | |  |
|  | | |  | | | Cough | | | 1.31 | | | (1.13, 1.53) | | | **** | | | | 1.20 | | | (1.02, 1.40) | | | *** | | | | 1.08 | | | (0.89, 1.31) | | | *0.43* | | | | 0.87 | | | (0.72, 1.05) | | | *0.15* | | | | | |  |
| *Maternal characteristics* | | | | | | | | |  | | |  | | |  | | | |  | | |  | | |  | | | |  | | |  | | |  | | | |  | | |  | | |  | | | | | |  |
|  | | | Age (years) | | | | | |  | | |  | | |  | | | |  | | |  | | |  | | | |  | | |  | | |  | | | |  | | |  | | |  | | | | | |  |
|  | | |  | | | 15-24 | | | 1.78 | | | (1.46, 2.17) | | | **** | | | | 1.74 | | | (1.41, 2.14) | | | **** | | | | 0.99 | | | (0.78, 1.25) | | | *0.91* | | | | 0.79 | | | (0.64, 0.99) | | | *** | | | | | |  |
|  | | |  | | | 25-34 | | | 1.00 | | | (Referent) | | |  | | | | 1.00 | | | (Referent) | | |  | | | | 1.00 | | | (Referent) | | |  | | | | 1.00 | | | (Referent) | | |  | | | | | |  |
|  | | |  | | | 35-49 | | | 0.67 | | | (0.55, 0.82) | | | **** | | | | 0.78 | | | (0.63, 0.95) | | | *** | | | | 0.96 | | | (0.73, 1.27) | | | *0.78* | | | | 0.90 | | | (0.69, 1.18) | | | *0.46* | | | | | |  |
|  | | | BMI (kg/m^2^) | | | | | |  | | |  | | |  | | | |  | | |  | | |  | | | |  | | |  | | |  | | | |  | | |  | | |  | | | | | |  |
|  | | |  | | | <18.5 | | | 1.04 | | | (0.89, 1.22) | | | *0.60* | | | | 1.10 | | | (0.93, 1.29) | | | *0.27* | | | | 0.97 | | | (0.79, 1.19) | | | *0.75* | | | | 1.07 | | | (0.86, 1.32) | | | *0.56* | | | | | |  |
|  | | |  | | | 18.5-24.9 | | | 1.00 | | | (Referent) | | |  | | | | 1.00 | | | (Referent) | | |  | | | | 1.00 | | | (Referent) | | |  | | | | 1.00 | | | (Referent) | | |  | | | | | |  |
|  | | |  | | | >=25 | | | 1.05 | | | (0.74, 1.48) | | | *0.79* | | | | 1.06 | | | (0.78, 1.43) | | | *0.73* | | | | 1.28 | | | (0.90, 1.82) | | | *0.18* | | | | 1.41 | | | (1.06, 1.87) | | | *** | | | | | |  |
|  | | | Reproductive health care | | | | | |  | | |  | | |  | | | |  | | |  | | |  | | | |  | | |  | | |  | | | |  | | |  | | |  | | | | | |  |
|  | | |  | | | Delivered at health facility | | | 1.54 | | | (1.22, 1.93) | | | **** | | | | 1.36 | | | (1.12, 1.66) | | | **** | | | | 1.77 | | | (1.42, 2.19) | | | **** | | | | 1.31 | | | (1.08, 1.59) | | | **** | | | | | |  |
|  | | |  | | | Type of delivery assistance | | |  | | |  | | |  | | | |  | | |  | | |  | | | |  | | |  | | |  | | | |  | | |  | | |  | | | | | |  |
|  | | |  | | | Health professional | | | 1.51 | | | (1.23, 1.86) | | | **** | | | | 1.39 | | | (1.15, 1.69) | | | **** | | | | 1.84 | | | (1.48, 2.28) | | | **** | | | | 1.45 | | | (1.19, 1.77) | | | **** | | | | | |  |
|  | | |  | | | Traditional birth attendant | | | 1.01 | | | (0.80, 1.27) | | | *0.94* | | | | 1.03 | | | (0.80, 1.33) | | | *0.82* | | | | 1.50 | | | (1.10, 2.03) | | | **** | | | | 1.05 | | | (0.77, 1.44) | | | *0.76* | | | | | |  |
|  | | |  | | | Other | | | 1.00 | | | (Referent) | | |  | | | | 1.00 | | | (Referent) | | |  | | | | 1.00 | | | (Referent) | | |  | | | | 1.00 | | | (Referent) | | |  | | | | | |  |
|  | | |  | | | Caesarean delivery | | | 1.90 | | | (1.33, 2.72) | | | **** | | | | 1.21 | | | (0.93, 1.57) | | | *0.16* | | | | 1.88 | | | (1.43, 2.46) | | | **** | | | | 1.34 | | | (1.07, 1.68) | | | **** | | | | | |  |
|  | | | | | | | | | | | | | | | | | | | | | | | | | | | | | | | | | | | | | | | | | | | | | | | | |  |  |  |
| **Supplemental Table 3 cont’** | | | | | | | | | | | | | | | | | | | | | | | | | | | | | | | | | | | | | | | | | | | | | | | | |  |  |  |
|  | |  | | |  | | | 2004 | | | | | | | | | 2007 | | | | | | | | | | 2011 | | | | | | | | | | 2014 | | | | | | | | | |  |  |  |  |  |
|  | |  | | |  | | | Estimate | | | | | | *P-value* | | | Estimate | | | | | | | *P-value* | | | Estimate | | | | | | | *P-value* | | | Estimate | | | | | | | *P-value* | | |  |  |  |  |  |
|  | |  | | |  | | | OR | | | (95%CI) | | |  |  |  | OR | | | | (95%CI) | | |  |  |  | OR | | | | (95%CI) | | |  |  |  | OR | | | | (95%CI) | | |  |  |  |  |  |  |  |  |
|  | | |  | | | Antenatal clinic visits | | |  | | |  | | |  | | | |  | | |  | | |  | | | |  | | |  | | |  | | | |  | | |  | | |  | | | | | |  |
|  | | |  | | | None | | | 0.70 | | | (0.59, 0.83) | | | **** | | | | 0.87 | | | (0.73, 1.04) | | | *0.12* | | | | 0.59 | | | (0.48, 0.74) | | | **** | | | | 0.77 | | | (0.60, 0.98) | | | *** | | | | | |  |
|  | | |  | | | 1-3 | | | 1.00 | | | (Referent) | | |  | | | | 1.00 | | | (Referent) | | |  | | | | 1.00 | | | (Referent) | | |  | | | | 1.00 | | | (Referent) | | |  | | | | | |  |
|  | | |  | | | ≥4 | | | 1.23 | | | (0.99, 1.53) | | | *0.06* | | | | 1.23 | | | (1.00, 1.51) | | | *** | | | | 1.27 | | | (1.00, 1.61) | | | *0.05* | | | | 1.21 | | | (0.98, 1.50) | | | *0.08* | | | | | |  |
|  | | |  | | | Postnatal check-up on woman | | |  | | |  | | |  | | | |  | | |  | | |  | | | |  | | |  | | |  | | | |  | | |  | | |  | | | | | |  |
|  | | |  | | | 0-1d | | | 1.00 | | | (Referent) | | |  | | | | 1.00 | | | (Referent) | | |  | | | | 1.00 | | | (Referent) | | |  | | | | 1.00 | | | (Referent) | | |  | | | | | |  |
|  | | |  | | | >=2d | | | 1.11 | | | (0.76, 1.61) | | | *0.59* | | | | 0.81 | | | (0.54, 1.21) | | | *0.30* | | | | 0.70 | | | (0.43, 1.15) | | | *0.16* | | | | 0.84 | | | (0.58, 1.21) | | | *0.34* | | | | | |  |
|  | | |  | | | Missing or unknown | | | 0.99 | | | (0.76, 1.28) | | | *0.93* | | | | 0.67 | | | (0.57, 0.80) | | | **** | | | | 0.56 | | | (0.44, 0.70) | | | **** | | | | 0.67 | | | (0.54, 0.81) | | | **** | | | | | |  |
|  | | |  | | | Postnatal check-up on child | | |  | | |  | | |  | | | |  | | |  | | |  | | | |  | | |  | | |  | | | |  | | |  | | |  | | | | | |  |
|  | | |  | | | 0-1d | | |  | | | - | | |  | | | | 1.00 | | | (Referent) | | |  | | | | 1.00 | | | (Referent) | | |  | | | | 1.00 | | | (Referent) | | |  | | | | | |  |
|  | | |  | | | >=2d | | |  | | | - | | |  | | | | 0.53 | | | (0.38, 0.75) | | | **** | | | | 0.95 | | | (0.70, 1.29) | | | *0.74* | | | | 0.98 | | | (0.72, 1.32) | | | *0.87* | | | | | |  |
|  | | |  | | | Missing or unknown | | |  | | | - | | |  | | | | 0.67 | | | (0.57, 0.80) | | | **** | | | | 0.70 | | | (0.57, 0.87) | | | **** | | | | 0.68 | | | (0.55, 0.84) | | | **** | | | | | |  |
|  | | | Maternal education | | | | | |  | | |  | | |  | | | |  | | |  | | |  | | | |  | | |  | | |  | | | |  | | |  | | |  | | | | | |  |
|  | | |  | | | No education | | | 0.61 | | | (0.51, 0.73) | | | **** | | | | 0.59 | | | (0.49, 0.72) | | | **** | | | | 0.45 | | | (0.35, 0.59) | | | **** | | | | 0.57 | | | (0.43, 0.75) | | | **** | | | | | |  |
|  | | |  | | | Primary | | | 0.68 | | | (0.56, 0.82) | | | **** | | | | 0.69 | | | (0.58, 0.82) | | | **** | | | | 0.58 | | | (0.47, 0.72) | | | **** | | | | 0.63 | | | (0.51, 0.78) | | | **** | | | | | |  |
|  | | |  | | | Secondary or higher | | | 1.00 | | | (Referent) | | |  | | | | 1.00 | | | (Referent) | | |  | | | | 1.00 | | | (Referent) | | |  | | | | 1.00 | | | (Referent) | | |  | | | | | |  |
|  | | | Exposure to media: at least once a week | | | | | |  | | |  | | |  | | | |  | | |  | | |  | | | |  | | |  | | |  | | | |  | | |  | | |  | | | | | |  |
|  | | |  | | | Reading newspaper | | | 1.48 | | | (1.11, 1.97) | | | **** | | | | 1.17 | | | (0.86, 1.59) | | | *0.31* | | | | 1.71 | | | (1.08, 2.70) | | | *** | | | | 1.40 | | | (0.93, 2.10) | | | *0.11* | | | | | |  |
|  | | |  | | | Listening to radio | | | 1.18 | | | (1.00, 1.38) | | | *** | | | | 1.05 | | | (0.86, 1.28) | | | *0.64* | | | | 1.27 | | | (0.80, 2.01) | | | *0.32* | | | | 1.88 | | | (0.98, 3.61) | | | *0.06* | | | | | |  |
|  | | |  | | | Watching TV | | | 1.22 | | | (1.05, 1.43) | | | *** | | | | 1.08 | | | (0.93, 1.26) | | | *0.33* | | | | 1.34 | | | (1.11, 1.64) | | | **** | | | | 1.47 | | | (1.21, 1.77) | | | **** | | | | | |  |
|  | | | Involved in decision making on | | | | | |  | | |  | | |  | | | |  | | |  | | |  | | | |  | | |  | | |  | | | |  | | |  | | |  | | | | | |  |
|  | | |  | | | How man's income is used | | |  | | | - | | |  | | | |  | | | - | | |  | | | |  | | | - | | |  | | | |  | | | - | | |  | | | | | |  |
|  | | |  | | | Large household purchases | | | 1.02 | | | (0.87, 1.20) | | | *0.80* | | | | 0.86 | | | (0.73, 1.01) | | | *0.07* | | | | 1.03 | | | (0.85, 1.25) | | | *0.76* | | | | 1.06 | | | (0.88, 1.27) | | | *0.56* | | | | | |  |
|  | | |  | | | Visiting family and friends | | | 0.88 | | | (0.75, 1.03) | | | *0.12* | | | | 0.93 | | | (0.79, 1.09) | | | *0.38* | | | | 0.96 | | | (0.79, 1.17) | | | *0.71* | | | | 1.08 | | | (0.90, 1.30) | | | *0.42* | | | | | |  |
|  | | |  | | | Regarding own health care | | | 0.91 | | | (0.78, 1.07) | | | *0.24* | | | | 0.88 | | | (0.75, 1.04) | | | *0.13* | | | | 1.01 | | | (0.83, 1.23) | | | *0.90* | | | | 1.10 | | | (0.91, 1.33) | | | *0.32* | | | | | |  |
|  | | | Appropriate attitude towards domestic violence: no queried situation was justified | | | | | |  | | | - | | |  | | | | 1.18 | | | (1.01, 1.39) | | | *** | | | | 1.04 | | | (0.85, 1.27) | | | *0.71* | | | | 1.07 | | | (0.87, 1.31) | | | *0.52* | | | | | |  |
|  | | | Women's empowerment score (5 items) | | | | | |  | | |  | | |  | | | |  | | |  | | |  | | | |  | | |  | | |  | | | |  | | |  | | |  | | | | | |  |
|  | | |  | | | <Weighted mean | | | 1.00 | | | (Referent) | | |  | | | | 1.00 | | | (Referent) | | |  | | | | 1.00 | | | (Referent) | | |  | | | | 1.00 | | | (Referent) | | |  | | | | | |  |
|  | | |  | | | >=Weighted mean | | | 1.25 | | | (0.98, 1.59) | | | *0.07* | | | | 1.26 | | | (0.95, 1.67) | | | *0.11* | | | | 0.92 | | | (0.76, 1.12) | | | *0.41* | | | | 1.08 | | | (0.90, 1.30) | | | *0.42* | | | | | |  |
| *Paternal characteristics* | | | | | | | | |  | | |  | | |  | | | |  | | |  | | |  | | | |  | | |  | | |  | | | |  | | |  | | |  | | | | | |  |
|  | | | Age (years) | | | | | |  | | |  | | |  | | | |  | | |  | | |  | | | |  | | |  | | |  | | | |  | | |  | | |  | | | | | |  |
|  | | |  | | | < 31 | | | 1.00 | | | (Referent) | | |  | | | | 1.00 | | | (Referent) | | |  | | | | 1.00 | | | (Referent) | | |  | | | | 1.00 | | | (Referent) | | |  | | | | | |  |
|  | | |  | | | >=31 | | | 0.67 | | | (0.57, 0.78) | | | **** | | | | 0.73 | | | (0.62, 0.86) | | | **** | | | | 1.12 | | | (0.92, 1.35) | | | *0.26* | | | | 1.22 | | | (1.01, 1.46) | | | *** | | | | | |  |
|  | | | Highest educational level | | | | | |  | | |  | | |  | | | |  | | |  | | |  | | | |  | | |  | | |  | | | |  | | |  | | |  | | | | | |  |
|  | | |  | | | No education | | | 0.72 | | | (0.60, 0.86) | | | **** | | | | 0.73 | | | (0.61, 0.88) | | | **** | | | | 0.45 | | | (0.36, 0.57) | | | **** | | | | 0.70 | | | (0.55, 0.89) | | | **** | | | | | |  |
|  | | |  | | | Primary | | | 0.78 | | | (0.64, 0.95) | | | *** | | | | 0.75 | | | (0.62, 0.90) | | | **** | | | | 0.76 | | | (0.61, 0.95) | | | *** | | | | 0.80 | | | (0.65, 0.99) | | | *** | | | | | |  |
|  | | |  | | | Secondary or higher | | | 1.00 | | | (Referent) | | |  | | | | 1.00 | | | (Referent) | | |  | | | | 1.00 | | | (Referent) | | |  | | | | 1.00 | | | (Referent) | | |  | | | | | |  |
| *Household characteristics* | | | | | | | | |  | | |  | | |  | | | |  | | |  | | |  | | | |  | | |  | | |  | | | |  | | |  | | |  | | | | | |  |
|  | | | Female household head | | | | | | 0.61 | | | (0.44, 0.85) | | | **** | | | | 0.96 | | | (0.73, 1.27) | | | *0.78* | | | | 0.85 | | | (0.60, 1.21) | | | *0.36* | | | | 0.97 | | | (0.71, 1.34) | | | *0.87* | | | | | |  |
|  | | | No. of HH members | | | | | |  | | |  | | |  | | | |  | | |  | | |  | | | |  | | |  | | |  | | | |  | | |  | | |  | | | | | |  |
|  | | |  | | | <Weighted mean (9.0) | | | 1.00 | | | (Referent) | | |  | | | | 1.00 | | | (Referent) | | |  | | | | 1.00 | | | (Referent) | | |  | | | | 1.00 | | | (Referent) | | |  | | | | | |  |
|  | | |  | | | >=Weighted mean (9.0) | | | 0.93 | | | (0.74, 1.17) | | | **** | | | | 0.84 | | | (0.65, 1.09) | | | **** | | | | 1.05 | | | (0.85, 1.28) | | | *0.66* | | | | 0.99 | | | (0.80, 1.22) | | | *0.94* | | | | | |  |
|  | | | | | | | | | | | | | | | | | | | | | | | | | | | | | | | | | | | | | | | | | | | | | | | | | | |  |
| **Supplemental Table 3 cont’** | | | | | | | | | | | | | | | | | | | | | | | | | | | | | | | | | | | | | | | | | | | | | | | | | | |  |
|  | | |  | | |  | | | 2004 | | | | | | | | | | 2007 | | | | | | | | | | 2011 | | | | | | | | | | 2014 | | | | | | | | | | |  |  |
|  | | |  | | |  | | | Estimate | | | | | | *P-value* | | | Estimate | | | | | | | *P-value* | | | Estimate | | | | | | | *P-value* | | | Estimate | | | | | | | *P-value* | | |  |  |  |  |
|  | | |  | | |  | | | OR | | | (95%CI) | | |  |  |  | OR | | | | (95%CI) | | |  |  |  | OR | | | | (95%CI) | | |  |  |  | OR | | | | (95%CI) | | |  |  |  |  |  |  |  |
|  | | | No. of children under 5 years | | | | | |  | | |  | | |  | | | |  | | |  | | |  | | | |  | | |  | | |  | | | |  | | |  | | |  | | | | | |  |
|  | | |  | | | <Weighted mean (2.3) | | | 1.00 | | | (Referent) | | |  | | | | 1.00 | | | (Referent) | | |  | | | | 1.00 | | | (Referent) | | |  | | | | 1.00 | | | (Referent) | | |  | | | | | |  |
|  | | |  | | | >=Weighted mean (2.3) | | | 0.70 | | | (0.56, 0.87) | | | **** | | | | 0.77 | | | (0.59, 0.99) | | | *** | | | | 0.80 | | | (0.66, 0.98) | | | *** | | | | 0.79 | | | (0.65, 0.96) | | | *** | | | | | |  |
|  | | | Type of cooking fuel | | | | | |  | | |  | | |  | | | |  | | |  | | |  | | | |  | | |  | | |  | | | |  | | |  | | |  | | | | | |  |
|  | | |  | | | Electricity, LPG, natural gas, biogas | | | 1.43 | | | (1.07, 1.92) | | | *** | | | | 1.20 | | | (0.91, 1.58) | | | *0.20* | | | | 1.50 | | | (1.06, 2.14) | | | *** | | | | 1.12 | | | (0.83, 1.52) | | | *0.46* | | | | | |  |
|  | | |  | | | Wood, straw/ shrubs/ grass, animal dung and other | | | 1.00 | | | (Referent) | | |  | | | | 1.00 | | | (Referent) | | |  | | | | 1.00 | | | (Referent) | | |  | | | | 1.00 | | | (Referent) | | |  | | | | | |  |
|  | | | Water source | | | | | |  | | |  | | |  | | | |  | | |  | | |  | | | |  | | |  | | |  | | | |  | | |  | | |  | | | | | |  |
|  | | |  | | | Unimproved source of drinking water | | | 1.15 | | | (0.77, 1.72) | | | *0.49* | | | | 1.20 | | | (0.80, 1.79) | | | *0.38* | | | | 0.89 | | | (0.42, 1.88) | | | *0.76* | | | | 1.18 | | | (0.66, 2.11) | | | *0.57* | | | | | |  |
|  | | |  | | | Source for water not in own dwelling or yard/plot | | |  | | | - | | |  | | | |  | | | - | | |  | | | | 0.73 | | | (0.59, 0.91) | | | **** | | | | 0.76 | | | (0.61, 0.94) | | | *** | | | | | |  |
|  | | |  | | | Time to get to water source  (min) | | |  | | |  | | |  | | | |  | | |  | | |  | | | |  | | |  | | |  | | | |  | | |  | | |  | | | | | |  |
|  | | |  | | | 0 | | |  | | | - | | |  | | | |  | | | - | | |  | | | | 1.00 | | | (Referent) | | | *0* | | | | 1.00 | | | (Referent) | | | *0* | | | | | |  |
|  | | |  | | | 1-59 | | |  | | | - | | |  | | | |  | | | - | | |  | | | | 0.72 | | | (0.58, 0.89) | | | **** | | | | 0.79 | | | (0.64, 0.98) | | | *** | | | | | |  |
|  | | |  | | | >=60 | | |  | | | - | | |  | | | |  | | | - | | |  | | | | 0.79 | | | (0.25, 2.53) | | | *0.70* | | | | 0.21 | | | (0.07, 0.59) | | | **** | | | | | |  |
|  | | | Toilet condition | | | | | |  | | |  | | |  | | | |  | | |  | | |  | | | |  | | |  | | |  | | | |  | | |  | | |  | | | | | |  |
|  | | |  | | | Unimproved toilet facility | | | 0.86 | | | (0.73, 1.00) | | | *0.05* | | | | 0.93 | | | (0.80, 1.09) | | | *0.39* | | | | 0.70 | | | (0.57, 0.84) | | | **** | | | | 0.92 | | | (0.75, 1.13) | | | *0.45* | | | | | |  |
|  | | |  | | | Shared toilet with other households | | |  | | | - | | |  | | | | 1.05 | | | (0.90, 1.23) | | | *0.54* | | | | 0.79 | | | (0.65, 0.97) | | | *** | | | | 0.79 | | | (0.65, 0.97) | | | *** | | | | | |  |
|  | | | HH wealth | | | | | |  | | |  | | |  | | | |  | | |  | | |  | | | |  | | |  | | |  | | | |  | | |  | | |  | | | | | |  |
|  | | |  | | | Richest | | | 1.00 | | | (Referent) | | |  | | | | 1.00 | | | (Referent) | | |  | | | | 1.00 | | | (Referent) | | |  | | | | 1.00 | | | (Referent) | | |  | | | | | |  |
|  | | |  | | | Richer | | | 0.67 | | | (0.52, 0.86) | | | **** | | | | 0.88 | | | (0.69, 1.12) | | | *0.29* | | | | 0.71 | | | (0.52, 0.96) | | | *** | | | | 0.89 | | | (0.66, 1.21) | | | *0.46* | | | | | |  |
|  | | |  | | | Middle | | | 0.75 | | | (0.59, 0.97) | | | *** | | | | 0.87 | | | (0.68, 1.11) | | | *0.26* | | | | 0.86 | | | (0.63, 1.19) | | | *0.38* | | | | 0.79 | | | (0.58, 1.07) | | | *0.13* | | | | | |  |
|  | | |  | | | Poorer | | | 0.59 | | | (0.46, 0.76) | | | **** | | | | 0.78 | | | (0.61, 0.98) | | | *** | | | | 0.61 | | | (0.44, 0.84) | | | **** | | | | 0.65 | | | (0.48, 0.90) | | | **** | | | | | |  |
|  | | |  | | | Poorest | | | 0.74 | | | (0.59, 0.94) | | | *** | | | | 0.71 | | | (0.56, 0.90) | | | **** | | | | 0.44 | | | (0.32, 0.60) | | | **** | | | | 0.59 | | | (0.43, 0.81) | | | **** | | | | | |  |
| *Community characteristics* | | | | | | | | |  | | |  | | |  | | | |  | | |  | | |  | | | |  | | |  | | |  | | | |  | | |  | | |  | | | | | |  |
|  | | | Rural residence | | | | | | 1.03 | | | (0.86, 1.23) | | | *0.77* | | | | 0.82 | | | (0.70, 0.96) | | | *** | | | | 0.73 | | | (0.57, 0.94) | | | *** | | | | 0.86 | | | (0.69, 1.08) | | | *0.19* | | | | | |  |
|  | | | Geographical region | | | | | |  | | |  | | |  | | | |  | | |  | | |  | | | |  | | |  | | |  | | | |  | | |  | | |  | | | | | |  |
|  | | |  | | | Barisal | | | 1.00 | | | (Referent) | | |  | | | | 1.00 | | | (Referent) | | |  | | | | 1.00 | | | (Referent) | | |  | | | | 1.00 | | | (Referent) | | |  | | | | | |  |
|  | | |  | | | Chittagong | | | 1.27 | | | (0.95, 1.71) | | | *0.11* | | | | 1.16 | | | (0.88, 1.54) | | | *0.29* | | | | 0.71 | | | (0.47, 1.05) | | | *0.09* | | | | 0.73 | | | (0.51, 1.05) | | | *0.09* | | | | | |  |
|  | | |  | | | Dhaka | | | 0.94 | | | (0.70, 1.25) | | | *0.67* | | | | 1.13 | | | (0.86, 1.48) | | | *0.38* | | | | 1.11 | | | (0.73, 1.68) | | | *0.62* | | | | 0.98 | | | (0.68, 1.40) | | | *0.90* | | | | | |  |
|  | | |  | | | Khulna | | | 0.98 | | | (0.72, 1.34) | | | *0.89* | | | | 1.23 | | | (0.90, 1.67) | | | *0.20* | | | | 2.76 | | | (1.69, 4.50) | | | **** | | | | 1.89 | | | (1.23, 2.89) | | | **** | | | | | |  |
|  | | |  | | | Rajshahi | | | 1.22 | | | (0.92, 1.63) | | | *0.17* | | | | 1.33 | | | (1.00, 1.77) | | | *** | | | | 1.25 | | | (0.85, 1.84) | | | *0.25* | | | | 1.35 | | | (0.95, 1.92) | | | *0.09* | | | | | |  |
|  | | |  | | | Sylhet | | | 0.70 | | | (0.50, 0.97) | | | *** | | | | 1.05 | | | (0.78, 1.40) | | | *0.76* | | | | 0.77 | | | (0.51, 1.18) | | | *0.23* | | | | 0.79 | | | (0.54, 1.15) | | | *0.22* | | | | | |  |
|  | | | Women completed primary or higher education | | | | | | 1.06 | | | (0.68, 1.66) | | | *0.32* | | | | 1.39 | | | (0.91, 2.14) | | | *0.13* | | | | 2.63 | | | (9.92, 0.00) | | | *0.28* | | | | 1.51 | | | (5.86, 0.00) | | | *0.15* | | | | | |  |
|  | | | Women's empowerment | | | | | | 1.17 | | | (0.97, 1.40) | | | *0.30* | | | | 1.18 | | | (1.02, 1.36) | | | *** | | | | 1.19 | | | (1.83, 0.00) | | | *0.33* | | | | 1.01 | | | (1.52, 0.04) | | | *0.15* | | | | | |  |
|  | | | Rank of access to health care | | | | | |  | | |  | | |  | | | |  | | |  | | |  | | | |  | | |  | | |  | | | |  | | |  | | |  | | | | | |  |
|  | | |  | | | Highest (best access) | | | 1.00 | | | (Referent) | | |  | | | | 1.00 | | | (Referent) | | |  | | | | 1.00 | | | (Referent) | | |  | | | | 1.00 | | | (Referent) | | |  | | | | | |  |
|  | | |  | | | Higher | | | 0.84 | | | (0.64, 1.09) | | | *0.19* | | | | 1.06 | | | (0.82, 1.37) | | | *0.65* | | | | 0.78 | | | (0.54, 1.14) | | | *0.20* | | | | 0.65 | | | (0.46, 0.91) | | | *** | | | | | |  |
|  | | |  | | | Medium | | | 0.88 | | | (0.68, 1.14) | | | *0.33* | | | | 0.92 | | | (0.71, 1.20) | | | *0.55* | | | | 0.76 | | | (0.53, 1.08) | | | *0.13* | | | | 0.60 | | | (0.44, 0.84) | | | **** | | | | | |  |
|  | | |  | | | Lower | | | 0.77 | | | (0.59, 1.01) | | | *0.06* | | | | 1.13 | | | (0.88, 1.45) | | | *0.33* | | | | 0.60 | | | (0.42, 0.86) | | | **** | | | | 0.62 | | | (0.45, 0.87) | | | **** | | | | | |  |
|  | | |  | | | Lowest (worse access) | | | 0.78 | | | (0.60, 1.01) | | | *0.06* | | | | 0.85 | | | (0.66, 1.09) | | | *0.20* | | | | 0.35 | | | (0.25, 0.50) | | | **** | | | | 0.53 | | | (0.39, 0.74) | | | **** | | | | | |  |
|  | | | Unimproved toilet | | | | | | 0.86 | | | (0.63, 1.17) | | | *0.31* | | | | 0.74 | | | (0.55, 1.00) | | | *** | | | | 0.25 | | | (0.59, 0.00) | | | *0.31* | | | | 0.45 | | | (1.01, 0.06) | | | *0.15* | | | | | |  |
|  | | | Share toilet with other households | | | | | |  | | | - | | |  | | | | 0.97 | | | (0.67, 1.42) | | | *0.88* | | | | 0.43 | | | (1.41, 0.41) | | | *0.37* | | | | 0.37 | | | (1.05, 0.07) | | | *0.15* | | | | | |  |
