# Supplementary material for: Stagnating trends in complementary feeding practices in Bangladesh: An analysis of national surveys from 2004‐2014
Source: Matern Child Nutr. 2018 Jul 12;14(Suppl 4):e12624. doi: 10.1111/mcn.12624 (PMC6586058; doi:10.1111/mcn.12624)
Supplement: Supplementary file 5 — Table S4: Factors [OR(95%CI)] in relation to MDD using year‐specific univariate multilevel logistic regression analysis [file MCN-14-e12624-s005.docx]

| **Supplemental Table 4:** Factors [OR(95%CI)] in relation to MDD using year-specific univariate multilevel logistic regression analysis | | | | | | | | |
| --- | --- | --- | --- | --- | --- | --- | --- | --- |
|  |  |  | 2011 | | | 2014 | | |
|  |  |  | Estimate | | *P-value* | Estimate | | *P-value* |
|  |  |  | OR | (95%CI) |  | OR | (95%CI) |  |
| *Child characteristics* | | |  |  |  |  |  |  |
|  | Female | | 1.13 | (0.93, 1.37) | *0.23* | 1.16 | (0.96, 1.39) | *0.13* |
|  | Age (months) | |  |  |  |  |  |  |
|  |  | 6-11 | 1.00 | (Referent) |  | 1.00 | (Referent) |  |
|  |  | 12-17 | 2.88 | (2.21, 3.74) | **** | 3.05 | (2.34, 3.97) | **** |
|  |  | 18-23 | 3.77 | (2.89, 4.91) | **** | 5.16 | (3.95, 6.73) | **** |
|  | Birth order | |  |  |  |  |  |  |
|  |  | Firstborn | 1.56 | (1.27, 1.91) | **** | 1.44 | (1.18, 1.74) | **** |
|  |  | Second to fourth | 1.00 | (Referent) |  | 1.00 | (Referent) |  |
|  |  | Fifth and more | 0.65 | (0.43, 1.00) | *** | 0.84 | (0.55, 1.29) | *0.43* |
|  | Birth interval (month) | |  |  |  |  |  |  |
|  |  | No previous birth | 1.72 | (1.40, 2.11) | **** | 1.48 | (1.21, 1.80) | **** |
|  |  | <24 | 1.31 | (0.90, 1.91) | *0.16* | 1.15 | (0.76, 1.72) | *0.51* |
|  |  | >=24 | 1.00 | (Referent) |  | 1.00 | (Referent) |  |
|  | Perceived birth weight | |  |  |  |  |  |  |
|  |  | Smaller than average | 0.80 | (0.61, 1.04) | *0.10* | 0.88 | (0.69, 1.13) | *0.31* |
|  |  | Average | 1.00 | (Referent) |  | 1.00 | (Referent) |  |
|  |  | Larger than average | 1.31 | (1.00, 1.73) | *0.05* | 1.30 | (0.99, 1.71) | *0.06* |
|  | Received vitamin A supplementation in the past 6 months | | 1.66 | (1.35, 2.04) | **** | 1.65 | (1.35, 2.01) | **** |
|  | Received iron pills, sprinkles or syrup in the last 7 days | | 1.13 | (0.62, 2.05) | *0.69* | 1.69 | (1.12, 2.54) | *** |
|  | Age-appropriate vaccination | |  |  |  |  |  |  |
|  |  | None | 0.54 | (0.25, 1.16) | *0.11* | 0.40 | (0.20, 0.81) | *** |
|  |  | Some | 0.59 | (0.44, 0.79) | **** | 0.52 | (0.40, 0.68) | **** |
|  |  | Complete | 1.00 | (Referent) |  | 1.00 | (Referent) |  |
|  | Child health: had the following symptom in the past 2 weeks | |  |  |  |  |  |  |
|  |  | Diarrhea | 0.68 | (0.46, 1.02) | *0.06* | 1.12 | (0.79, 1.60) | *0.51* |
|  |  | Fever | 0.81 | (0.66, 0.99) | *** | 0.79 | (0.65, 0.95) | *** |
|  |  | Cough | 0.84 | (0.69, 1.03) | *0.09* | 0.85 | (0.70, 1.03) | *0.10* |
| *Maternal characteristics* | | |  |  |  |  |  |  |
|  | Age (years) | |  |  |  |  |  |  |
|  |  | 15-24 | 1.04 | (0.82, 1.33) | *0.75* | 0.99 | (0.78, 1.24) | *0.91* |
|  |  | 25-34 | 1.00 | (Referent) |  | 1.00 | (Referent) |  |
|  |  | 35-49 | 0.83 | (0.61, 1.11) | *0.21* | 1.14 | (0.87, 1.50) | *0.33* |
|  | BMI (kg/m^2^) | |  |  |  |  |  |  |
|  |  | <18.5 | 0.73 | (0.58, 0.91) | **** | 0.87 | (0.70, 1.09) | *0.23* |
|  |  | 18.5-24.9 | 1.00 | (Referent) |  | 1.00 | (Referent) |  |
|  |  | >=25 | 1.71 | (1.24, 2.35) | **** | 1.46 | (1.12, 1.90) | **** |
|  | Reproductive health care | |  |  |  |  |  |  |
|  |  | Delivered at health facility | 2.26 | (1.84, 2.77) | **** | 1.60 | (1.32, 1.94) | **** |
|  |  | Type of delivery assistance |  |  |  |  |  |  |
|  |  | Health professional | 2.36 | (1.91, 2.92) | **** | 1.75 | (1.43, 2.14) | **** |
|  |  | Traditional birth attendant | 1.38 | (1.00, 1.90) | *** | 0.97 | (0.68, 1.38) | *0.87* |
|  |  | Other | 1.00 | (Referent) |  | 1.00 | (Referent) |  |
|  |  | Caesarean delivery | 2.56 | (2.02, 3.25) | **** | 1.73 | (1.39, 2.14) | **** |
|  | | | | | | | | |
| **Supplemental Table 4 cont’** | | | | | | | | |
|  |  |  | 2011 | | | 2014 | | |
|  |  |  | Estimate | | *P-value* | Estimate | | *P-value* |
|  |  |  | OR | (95%CI) |  | OR | (95%CI) |  |
|  |  | Antenatal clinic visits |  |  |  |  |  |  |
|  |  | None | 0.69 | (0.54, 0.89) | **** | 0.63 | (0.48, 0.83) | **** |
|  |  | 1-3 | 1.00 | (Referent) |  | 1.00 | (Referent) |  |
|  |  | ≥4 | 1.91 | (1.52, 2.39) | **** | 1.38 | (1.12, 1.70) | **** |
|  |  | Postnatal check-up on woman |  |  |  |  |  |  |
|  |  | 0-1d | 1.00 | (Referent) |  | 1.00 | (Referent) |  |
|  |  | >=2d | 0.66 | (0.41, 1.07) | *0.09* | 1.17 | (0.81, 1.68) | *0.40* |
|  |  | Missing or unknown | 0.43 | (0.35, 0.53) | **** | 0.70 | (0.57, 0.87) | **** |
|  |  | Postnatal check-up on child |  |  |  |  |  |  |
|  |  | 0-1d | 1.00 | (Referent) | *0* | 1.00 | (Referent) | *0* |
|  |  | >=2d | 0.83 | (0.61, 1.12) | *0.21* | 0.96 | (0.71, 1.30) | *0.78* |
|  |  | Missing or unknown | 0.50 | (0.41, 0.63) | **** | 0.74 | (0.60, 0.92) | **** |
|  | Maternal education | |  |  |  |  |  |  |
|  |  | No education | 0.27 | (0.19, 0.38) | **** | 0.35 | (0.25, 0.49) | **** |
|  |  | Primary | 0.49 | (0.39, 0.61) | **** | 0.46 | (0.37, 0.58) | **** |
|  |  | Secondary or higher | 1.00 | (Referent) |  | 1.00 | (Referent) |  |
|  | Exposure to media: at least once a week | |  |  |  |  |  |  |
|  |  | Reading newspaper | 3.53 | (2.41, 5.18) | **** | 3.46 | (2.40, 4.99) | **** |
|  |  | Listening to radio | 1.00 | (0.63, 1.60) | *1.00* | 1.74 | (1.00, 3.02) | *** |
|  |  | Watching TV | 2.27 | (1.85, 2.78) | **** | 1.55 | (1.28, 1.87) | **** |
|  | Involved in decision making on | |  |  |  |  |  |  |
|  |  | How man's income is used |  | - |  |  | - |  |
|  |  | Large household purchases | 1.43 | (1.17, 1.74) | **** | 1.00 | (0.83, 1.21) | *1.00* |
|  |  | Visiting family and friends | 1.39 | (1.14, 1.71) | **** | 1.09 | (0.90, 1.32) | *0.37* |
|  |  | Regarding own health care | 1.43 | (1.17, 1.76) | **** | 1.17 | (0.97, 1.42) | *0.11* |
|  | Appropriate attitude towards domestic violence: no queried situation was justified | | 1.11 | (0.90, 1.37) | *0.34* | 1.22 | (0.99, 1.51) | *0.06* |
|  | Women's empowerment score (5 items) | |  |  |  |  |  |  |
|  |  | <Weighted mean | 1.00 | (Referent) |  | 1.00 | (Referent) |  |
|  |  | >=Weighted mean | 1.46 | (1.19, 1.78) | **** | 1.09 | (0.91, 1.32) | *0.35* |
| *Paternal characteristics* | | |  |  |  |  |  |  |
|  | Age (years) | |  |  |  |  |  |  |
|  |  | < 31 | 1.00 | (Referent) |  | 1.00 | (Referent) |  |
|  |  | >=31 | 1.10 | (0.90, 1.35) | *0.33* | 1.01 | (0.83, 1.21) | *0.95* |
|  | Highest educational level | |  |  |  |  |  |  |
|  |  | No education | 0.29 | (0.22, 0.38) | **** | 0.49 | (0.37, 0.63) | **** |
|  |  | Primary | 0.53 | (0.42, 0.67) | **** | 0.63 | (0.50, 0.78) | **** |
|  |  | Secondary or higher | 1.00 | (Referent) |  | 1.00 | (Referent) |  |
| *Household characteristics* | | |  |  |  |  |  |  |
|  | Female household head | | 1.38 | (0.97, 1.97) | *0.07* | 1.09 | (0.78, 1.51) | *0.62* |
|  | No. of HH members | |  |  |  |  |  |  |
|  |  | <Weighted mean (9.0) | 1.00 | (Referent) |  | 1.00 | (Referent) |  |
|  |  | >=Weighted mean (9.0) | 1.09 | (0.88, 1.34) | 0.42 | 1.04 | (0.86, 1.27) | 0.66 |
|  | | | | | | | | |
| **Supplemental Table 4 cont’** | | | | | | | | |
|  |  |  | 2011 | | | 2014 | | |
|  |  |  | Estimate | | *P-value* | Estimate | | *P-value* |
|  |  |  | OR | (95%CI) |  | OR | (95%CI) |  |
|  | No. of children under 5 years | |  |  |  |  |  |  |
|  |  | <Weighted mean (2.3) | 1.00 | (Referent) |  | 1.00 | (Referent) |  |
|  |  | >=Weighted mean (2.3) | 0.62 | (0.50, 0.77) | **** | 0.80 | (0.65, 0.98) | *** |
|  | Type of cooking fuel | |  |  |  |  |  |  |
|  |  | Electricity, LPG, natural gas, biogas | 2.16 | (1.60, 2.92) | **** | 1.51 | (1.12, 2.03) | **** |
|  |  | Wood, straw/ shrubs/ grass, animal dung and other | 1.00 | (Referent) |  | 1.00 | (Referent) |  |
|  | Water source | |  |  |  |  |  |  |
|  |  | Unimproved source of drinking water | 0.43 | (0.17, 1.10) | *0.08* | 0.76 | (0.41, 1.39) | *0.37* |
|  |  | Source for water not in own dwelling or yard/plot | 0.75 | (0.59, 0.94) | *** | 0.69 | (0.55, 0.87) | **** |
|  |  | Time to get to water source  (min) |  |  |  |  |  |  |
|  |  | 0 | 1.00 | (Referent) | *0* | 1.00 | (Referent) | *0* |
|  |  | 1-59 | 0.74 | (0.59, 0.93) | **** | 0.69 | (0.55, 0.86) | **** |
|  |  | >=60 | 0.48 | (0.10, 2.29) | *0.35* | 0.44 | (0.12, 1.59) | *0.21* |
|  | Toilet condition | |  |  |  |  |  |  |
|  |  | Unimproved toilet facility | 0.43 | (0.35, 0.53) | **** | 0.59 | (0.48, 0.74) | **** |
|  |  | Shared toilet with other households | 0.68 | (0.54, 0.84) | **** | 0.87 | (0.70, 1.07) | *0.18* |
|  | HH wealth | |  |  |  |  |  |  |
|  |  | Richest | 1.00 | (Referent) |  | 1.00 | (Referent) |  |
|  |  | Richer | 0.70 | (0.52, 0.92) | *** | 0.88 | (0.66, 1.17) | *0.38* |
|  |  | Middle | 0.45 | (0.33, 0.61) | **** | 0.61 | (0.45, 0.83) | **** |
|  |  | Poorer | 0.29 | (0.21, 0.40) | **** | 0.51 | (0.37, 0.70) | **** |
|  |  | Poorest | 0.19 | (0.14, 0.27) | **** | 0.39 | (0.28, 0.54) | **** |
| *Community characteristics* | | |  |  |  |  |  |  |
|  | Rural residence | | 0.50 | (0.40, 0.62) | **** | 0.74 | (0.59, 0.91) | **** |
|  | Geographical region | |  |  |  |  |  |  |
|  |  | Barisal | 1.00 | (Referent) |  | 1.00 | (Referent) |  |
|  |  | Chittagong | 0.90 | (0.60, 1.35) | *0.62* | 0.90 | (0.61, 1.31) | *0.58* |
|  |  | Dhaka | 1.13 | (0.75, 1.70) | *0.57* | 1.00 | (0.69, 1.46) | *0.99* |
|  |  | Khulna | 1.61 | (1.05, 2.48) | *** | 1.25 | (0.83, 1.88) | *0.28* |
|  |  | Rajshahi | 1.34 | (0.92, 1.95) | *0.13* | 1.10 | (0.77, 1.57) | *0.58* |
|  |  | Sylhet | 0.61 | (0.39, 0.96) | *** | 0.70 | (0.46, 1.05) | *0.08* |
|  | Women completed primary or higher education | | 5.93 | (23.48, 0.00) | *0.08* | 2.15 | (8.77, 0.00) | *0.08* |
|  | Women's empowerment | | 1.15 | (1.76, 0.00) | *0.17* | 1.12 | (1.68, 0.00) | *0.10* |
|  | Rank of access to health care | |  |  |  |  |  |  |
|  |  | Highest (best access) | 1.00 | (Referent) |  | 1.00 | (Referent) |  |
|  |  | Higher | 0.56 | (0.41, 0.77) | **** | 0.80 | (0.58, 1.10) | *0.17* |
|  |  | Medium | 0.48 | (0.35, 0.65) | **** | 0.67 | (0.49, 0.92) | *** |
|  |  | Lower | 0.31 | (0.22, 0.42) | **** | 0.74 | (0.54, 1.02) | *0.06* |
|  |  | Lowest (worse access) | 0.27 | (0.20, 0.38) | **** | 0.57 | (0.42, 0.79) | **** |
|  | Unimproved toilet | | 0.14 | (0.33, 0.00) | *0.09* | 0.27 | (0.62, 0.00) | *0.07* |
|  | Share toilet with other households | | 0.37 | (1.19, 0.17) | *0.20* | 0.32 | (0.93, 0.03) | *0.11* |
