# Supplementary material for: Stagnating trends in complementary feeding practices in Bangladesh: An analysis of national surveys from 2004‐2014
Source: Matern Child Nutr. 2018 Jul 12;14(Suppl 4):e12624. doi: 10.1111/mcn.12624 (PMC6586058; doi:10.1111/mcn.12624)
Supplement: Supplementary file 6 — Table S5: Factors [OR(95%CI)] in relation to MAD using year‐specific univariate multilevel logistic regression analysis [file MCN-14-e12624-s006.docx]

| **Supplemental Table 5:** Factors [OR(95%CI)] in relation to MAD using year-specific univariate multilevel logistic regression analysis | | | | | | | | | | | | | | | | | |
| --- | --- | --- | --- | --- | --- | --- | --- | --- | --- | --- | --- | --- | --- | --- | --- | --- | --- |
|  |  | |  | | | | 2011 | | | | | 2014 | | | | | |
|  |  | |  | | | | Estimate | | | *P-value* | | Estimate | | | | *P-value* | |
|  |  | |  | | | | OR | (95%CI) | |  |  | OR | | (95%CI) | |  |  |
| *Child characteristics* | | | | | | |  |  | |  | |  | |  | |  | |
|  | Female | | | | | | 1.05 | (0.85, 1.29) | | *0.66* | | 1.12 | | (0.91, 1.36) | | *0.28* | |
|  | Age (months) | | | | | |  |  | |  | |  | |  | |  | |
|  |  | | 6-11 | | | | 1.00 | (Referent) | |  | | 1.00 | | (Referent) | |  | |
|  |  | | 12-17 | | | | 2.84 | (2.14, 3.76) | | **** | | 3.04 | | (2.30, 4.01) | | **** | |
|  |  | | 18-23 | | | | 3.43 | (2.58, 4.56) | | **** | | 4.24 | | (3.21, 5.60) | | **** | |
|  | Birth order | | | | | |  |  | |  | |  | |  | |  | |
|  |  | | Firstborn | | | | 1.68 | (1.35, 2.09) | | **** | | 1.34 | | (1.09, 1.64) | | **** | |
|  |  | | Second to fourth | | | | 1.00 | (Referent) | |  | | 1.00 | | (Referent) | |  | |
|  |  | | Fifth and more | | | | 0.79 | (0.51, 1.23) | | *0.29* | | 0.84 | | (0.54, 1.33) | | *0.46* | |
|  | Birth interval (month) | | | | | |  |  | |  | |  | |  | |  | |
|  |  | | No previous birth | | | | 1.87 | (1.50, 2.34) | | **** | | 1.39 | | (1.13, 1.71) | | **** | |
|  |  | | <24 | | | | 1.60 | (1.09, 2.37) | | *** | | 1.27 | | (0.83, 1.93) | | *0.27* | |
|  |  | | >=24 | | | | 1.00 | (Referent) | |  | | 1.00 | | (Referent) | |  | |
|  | Perceived birth weight | | | | | |  |  | |  | |  | |  | |  | |
|  |  | | Smaller than average | | | | 0.78 | (0.58, 1.04) | | *0.09* | | 0.86 | | (0.66, 1.12) | | *0.26* | |
|  |  | | Average | | | | 1.00 | (Referent) | |  | | 1.00 | | (Referent) | |  | |
|  |  | | Larger than average | | | | 1.27 | (0.95, 1.70) | | *0.10* | | 1.12 | | (0.84, 1.50) | | *0.44* | |
|  | Received vitamin A supplementation in the past 6 months | | | | | | 1.57 | (1.26, 1.95) | | **** | | 1.80 | | (1.45, 2.22) | | **** | |
|  | Received iron pills, sprinkles or syrup in the last 7 days | | | | | | 1.23 | (0.66, 2.29) | | *0.52* | | 1.47 | | (0.95, 2.26) | | *0.08* | |
|  | Age-appropriate vaccination | | | | | |  |  | |  | |  | |  | |  | |
|  |  | | None | | | | 0.52 | (0.23, 1.18) | | *0.12* | | 0.33 | | (0.15, 0.74) | | **** | |
|  |  | | Some | | | | 0.61 | (0.44, 0.83) | | **** | | 0.51 | | (0.38, 0.69) | | **** | |
|  |  | | Complete | | | | 1.00 | (Referent) | |  | | 1.00 | | (Referent) | |  | |
|  | Child health: had the following symptom in the past 2 weeks | | | | | |  |  | |  | |  | |  | |  | |
|  |  | | Diarrhea | | | | 0.66 | (0.43, 1.02) | | *0.06* | | 1.28 | | (0.89, 1.84) | | *0.19* | |
|  |  | | Fever | | | | 0.85 | (0.69, 1.05) | | *0.13* | | 0.73 | | (0.60, 0.90) | | **** | |
|  |  | | Cough | | | | 0.84 | (0.68, 1.05) | | *0.12* | | 0.81 | | (0.66, 1.00) | | *** | |
| *Maternal characteristics* | | | | | | |  |  | |  | |  | |  | |  | |
|  | Age (years) | | | | | |  |  | |  | |  | |  | |  | |
|  |  | | 15-24 | | | | 1.08 | (0.84, 1.39) | | *0.56* | | 0.89 | | (0.70, 1.14) | | *0.36* | |
|  |  | | 25-34 | | | | 1.00 | (Referent) | |  | | 1.00 | | (Referent) | |  | |
|  |  | | 35-49 | | | | 0.79 | (0.57, 1.09) | | *0.15* | | 1.15 | | (0.86, 1.53) | | *0.34* | |
|  | BMI (kg/m^2^) | | | | | |  |  | |  | |  | |  | |  | |
|  |  | | <18.5 | | | | 0.79 | (0.62, 1.00) | | *0.05* | | 0.89 | | (0.71, 1.13) | | *0.34* | |
|  |  | | 18.5-24.9 | | | | 1.00 | (Referent) | |  | | 1.00 | | (Referent) | |  | |
|  |  | | >=25 | | | | 1.54 | (1.10, 2.17) | | *** | | 1.52 | | (1.15, 1.99) | | **** | |
|  | Reproductive health care | | | | | |  |  | |  | |  | |  | |  | |
|  |  | | Delivered at health facility | | | | 2.09 | (1.69, 2.59) | | **** | | 1.61 | | (1.31, 1.97) | | **** | |
|  |  | | Type of delivery assistance | | | |  |  | |  | |  | |  | |  | |
|  |  | | Health professional | | | | 2.18 | (1.74, 2.73) | | **** | | 1.81 | | (1.46, 2.24) | | **** | |
|  |  | | Traditional birth attendant | | | | 1.40 | (1.00, 1.97) | | *0.05* | | 1.05 | | (0.73, 1.52) | | *0.79* | |
|  |  | | Other | | | | 1.00 | (Referent) | |  | | 1.00 | | (Referent) | |  | |
|  |  | | Caesarean delivery | | | | 2.52 | (1.97, 3.22) | | **** | | 1.86 | | (1.49, 2.33) | | **** | |
|  | | | | | | | | | | | | | | | | |  |
| **Supplemental Table 5 cont’** | | | | | | | | | | | | | | | | |  |
|  | |  | |  | 2011 | | | | | | 2014 | | | | | |  |
|  | |  | |  | Estimate | | | | *P-value* | | Estimate | | | | *P-value* | |  |
|  | |  | |  | OR | (95%CI) | | |  |  | OR | | (95%CI) | |  |  |  |
|  |  | | Antenatal clinic visits | | | |  |  | |  | |  | |  | |  | |
|  |  | | None | | | | 0.75 | (0.58, 0.99) | | *** | | 0.58 | | (0.43, 0.78) | | **** | |
|  |  | | 1-3 | | | | 1.00 | (Referent) | |  | | 1.00 | | (Referent) | |  | |
|  |  | | ≥4 | | | | 1.70 | (1.34, 2.16) | | **** | | 1.36 | | (1.10, 1.69) | | **** | |
|  |  | | Postnatal check-up on woman | | | |  |  | |  | |  | |  | |  | |
|  |  | | 0-1d | | | | 1.00 | (Referent) | |  | | 1.00 | | (Referent) | |  | |
|  |  | | >=2d | | | | 0.67 | (0.40, 1.12) | | *0.13* | | 1.05 | | (0.71, 1.55) | | *0.80* | |
|  |  | | Missing or unknown | | | | 0.47 | (0.38, 0.59) | | **** | | 0.68 | | (0.54, 0.86) | | **** | |
|  |  | | Postnatal check-up on child | | | |  |  | |  | |  | |  | |  | |
|  |  | | 0-1d | | | | 1.00 | (Referent) | |  | | 1.00 | | (Referent) | |  | |
|  |  | | >=2d | | | | 0.80 | (0.58, 1.11) | | *0.18* | | 0.99 | | (0.72, 1.35) | | *0.94* | |
|  |  | | Missing or unknown | | | | 0.57 | (0.45, 0.71) | | **** | | 0.68 | | (0.54, 0.86) | | **** | |
|  | Maternal education | | | | | |  |  | |  | |  | |  | |  | |
|  |  | | No education | | | | 0.29 | (0.20, 0.43) | | **** | | 0.35 | | (0.24, 0.51) | | **** | |
|  |  | | Primary | | | | 0.55 | (0.44, 0.71) | | **** | | 0.48 | | (0.38, 0.62) | | **** | |
|  |  | | Secondary or higher | | | | 1.00 | (Referent) | |  | | 1.00 | | (Referent) | |  | |
|  | Exposure to media: at least once a week | | | | | |  |  | |  | |  | |  | |  | |
|  |  | | Reading newspaper | | | | 2.56 | (1.73, 3.79) | | **** | | 3.48 | | (2.41, 5.03) | | **** | |
|  |  | | Listening to radio | | | | 0.96 | (0.58, 1.59) | | *0.89* | | 2.18 | | (1.25, 3.81) | | **** | |
|  |  | | Watching TV | | | | 2.01 | (1.62, 2.49) | | **** | | 1.64 | | (1.34, 2.01) | | **** | |
|  | Involved in decision making on | | | | | |  |  | |  | |  | |  | |  | |
|  |  | | How man's income is used | | | |  | - | |  | |  | | - | |  | |
|  |  | | Large household purchases | | | | 1.43 | (1.15, 1.77) | | **** | | 0.97 | | (0.79, 1.18) | | *0.74* | |
|  |  | | Visiting family and friends | | | | 1.37 | (1.10, 1.71) | | **** | | 1.03 | | (0.84, 1.26) | | *0.76* | |
|  |  | | Regarding own health care | | | | 1.38 | (1.11, 1.72) | | **** | | 1.12 | | (0.91, 1.37) | | *0.28* | |
|  | Appropriate attitude towards domestic violence: no queried situation was justified | | | | | | 1.02 | (0.81, 1.27) | | *0.87* | | 1.20 | | (0.96, 1.50) | | *0.11* | |
|  | Women's empowerment score (5 items) | | | | | |  |  | |  | |  | |  | |  | |
|  |  | | <Weighted mean | | | | 1.00 | (Referent) | |  | | 1.00 | | (Referent) | |  | |
|  |  | | >=Weighted mean | | | | 1.44 | (1.16, 1.79) | | **** | | 1.02 | | (0.84, 1.24) | | *0.85* | |
| *Paternal characteristics* | | | | | | |  |  | |  | |  | |  | |  | |
|  | Age (years) | | | | | |  |  | |  | |  | |  | |  | |
|  |  | | < 31 | | | | 1.00 | (Referent) | |  | | 1.00 | | (Referent) | |  | |
|  |  | | >=31 | | | | 1.01 | (0.82, 1.25) | | *0.89* | | 1.12 | | (0.92, 1.36) | | *0.28* | |
|  | Highest educational level | | | | | |  |  | |  | |  | |  | |  | |
|  |  | | No education | | | | 0.31 | (0.23, 0.42) | | **** | | 0.48 | | (0.36, 0.63) | | **** | |
|  |  | | Primary | | | | 0.57 | (0.45, 0.73) | | **** | | 0.62 | | (0.49, 0.79) | | **** | |
|  |  | | Secondary or higher | | | | 1.00 | (Referent) | |  | | 1.00 | | (Referent) | |  | |
| *Household characteristics* | | | | | | |  |  | |  | |  | |  | |  | |
|  | Female household head | | | | | | 0.90 | (0.60, 1.35) | | *0.60* | | 1.02 | | (0.72, 1.45) | | *0.90* | |
|  | No. of HH members | | | | | |  |  | |  | |  | |  | |  | |
|  |  | | <Weighted mean (9.0) | | | | 1.00 | (Referent) | |  | | 1.00 | | (Referent) | |  | |
|  |  | | >=Weighted mean (9.0) | | | | 1.09 | (0.87, 1.36) | | 0.46 | | 0.99 | | (0.81, 1.20) | | 0.89 | |
|  | | | | | | | | | | | | | | | | |  |
| **Supplemental Table 5 cont’** | | | | | | | | | | | | | | | | |  |
|  | |  | |  | 2011 | | | | | | 2014 | | | | | |  |
|  | |  | |  | Estimate | | | | *P-value* | | Estimate | | | | *P-value* | |  |
|  | |  | |  | OR | (95%CI) | | |  |  | OR | | (95%CI) | |  |  |  |
|  | No. of children under 5 years | | | | | |  |  | |  | |  | |  | |  | |
|  |  | | <Weighted mean (2.3) | | | | 1.00 | (Referent) | |  | | 1.00 | | (Referent) | |  | |
|  |  | | >=Weighted mean (2.3) | | | | 0.61 | (0.48, 0.76) | | **** | | 0.81 | | (0.65, 1.00) | | *0.05* | |
|  | Type of cooking fuel | | | | | |  |  | |  | |  | |  | |  | |
|  |  | | Electricity, LPG, natural gas, biogas | | | | 1.88 | (1.36, 2.60) | | **** | | 1.57 | | (1.15, 2.15) | | **** | |
|  |  | | Wood, straw/ shrubs/ grass, animal dung and other | | | | 1.00 | (Referent) | |  | | 1.00 | | (Referent) | |  | |
|  | Water source | | | | | |  |  | |  | |  | |  | |  | |
|  |  | | Unimproved source of drinking water | | | | 0.57 | (0.23, 1.44) | | *0.24* | | 0.81 | | (0.43, 1.53) | | *0.52* | |
|  |  | | Source for water not in own dwelling or yard/plot | | | | 0.75 | (0.58, 0.96) | | *** | | 0.67 | | (0.52, 0.85) | | **** | |
|  |  | | Time to get to water source  (min) | | | |  |  | |  | |  | |  | |  | |
|  |  | | 0 | | | | 1.00 | (Referent) | |  | | 1.00 | | (Referent) | |  | |
|  |  | | 1-59 | | | | 0.74 | (0.58, 0.94) | | *** | | 0.66 | | (0.52, 0.84) | | **** | |
|  |  | | >=60 | | | | 0.62 | (0.13, 2.93) | | *0.54* | | 0.35 | | (0.08, 1.57) | | *0.17* | |
|  | Toilet condition | | | | | |  |  | |  | |  | |  | |  | |
|  |  | | Unimproved toilet facility | | | | 0.47 | (0.38, 0.59) | | **** | | 0.64 | | (0.51, 0.80) | | **** | |
|  |  | | Shared toilet with other households | | | | 0.71 | (0.57, 0.90) | | **** | | 0.77 | | (0.62, 0.97) | | *** | |
|  | HH wealth | | | | | |  |  | |  | |  | |  | |  | |
|  |  | | Richest | | | | 1.00 | (Referent) | |  | | 1.00 | | (Referent) | |  | |
|  |  | | Richer | | | | 0.74 | (0.55, 1.00) | | *** | | 0.85 | | (0.64, 1.15) | | *0.30* | |
|  |  | | Middle | | | | 0.57 | (0.42, 0.78) | | **** | | 0.56 | | (0.41, 0.78) | | **** | |
|  |  | | Poorer | | | | 0.33 | (0.24, 0.47) | | **** | | 0.47 | | (0.34, 0.66) | | **** | |
|  |  | | Poorest | | | | 0.25 | (0.17, 0.36) | | **** | | 0.37 | | (0.26, 0.52) | | **** | |
| *Community characteristics* | | | | | | |  |  | |  | |  | |  | |  | |
|  | Rural residence | | | | | | 0.56 | (0.45, 0.71) | | **** | | 0.71 | | (0.57, 0.88) | | **** | |
|  | Geographical region | | | | | |  |  | |  | |  | |  | |  | |
|  |  | | Barisal | | | | 1.00 | (Referent) | |  | | 1.00 | | (Referent) | |  | |
|  |  | | Chittagong | | | | 0.95 | (0.62, 1.46) | | *0.83* | | 0.80 | | (0.54, 1.19) | | *0.27* | |
|  |  | | Dhaka | | | | 1.48 | (0.96, 2.28) | | *0.07* | | 1.04 | | (0.71, 1.54) | | *0.82* | |
|  |  | | Khulna | | | | 2.14 | (1.37, 3.34) | | **** | | 1.34 | | (0.88, 2.03) | | *0.17* | |
|  |  | | Rajshahi | | | | 1.52 | (1.02, 2.27) | | *** | | 1.18 | | (0.82, 1.70) | | *0.37* | |
|  |  | | Sylhet | | | | 0.63 | (0.39, 1.02) | | *0.06* | | 0.60 | | (0.39, 0.93) | | *** | |
|  | Women completed primary or higher education | | | | | | 3.91 | (16.37, 0.00) | | *0.06* | | 2.21 | | (9.80, 0.00) | | *0.08* | |
|  | Women's empowerment | | | | | | 1.17 | (1.81, 0.00) | | *0.11* | | 1.11 | | (1.70, 0.00) | | *0.09* | |
|  | Rank of access to health care | | | | | |  |  | |  | |  | |  | |  | |
|  |  | | Highest (best access) | | | | 1.00 | (Referent) | |  | | 1.00 | | (Referent) | |  | |
|  |  | | Higher | | | | 0.67 | (0.48, 0.92) | | *** | | 0.73 | | (0.52, 1.01) | | *0.06* | |
|  |  | | Medium | | | | 0.53 | (0.39, 0.74) | | **** | | 0.62 | | (0.45, 0.86) | | **** | |
|  |  | | Lower | | | | 0.34 | (0.24, 0.48) | | **** | | 0.72 | | (0.52, 0.99) | | *** | |
|  |  | | Lowest (worse access) | | | | 0.31 | (0.22, 0.44) | | **** | | 0.52 | | (0.37, 0.73) | | **** | |
|  | Unimproved toilet | | | | | | 0.17 | (0.41, 0.00) | | *0.06* | | 0.26 | | (0.64, 0.00) | | *0.07* | |
|  | Share toilet with other households | | | | | | 0.44 | (1.48, 0.49) | | *0.14* | | 0.31 | | (0.95, 0.03) | | *0.10* | |
